# Inhibition profile of three biological nitrification inhibitors and their response to soil pH modification in two contrasting soils

**DOI:** 10.1093/femsec/fiae072

**Published:** 2024-05-03

**Authors:** Paula A Rojas-Pinzon, Judith Prommer, Christopher J Sedlacek, Taru Sandén, Heide Spiegel, Petra Pjevac, Lucia Fuchslueger, Andrew T Giguere

**Affiliations:** Centre for Microbiology and Environmental Systems Science, Department for Microbiology and Ecosystem Science, University of Vienna, Djerassiplatz 1, 1030, Vienna, Austria; Doctoral School in Microbiology and Environmental Science, University of Vienna, Djerassiplatz 1, 1030, Vienna, Austria; Centre for Microbiology and Environmental Systems Science, Department for Microbiology and Ecosystem Science, University of Vienna, Djerassiplatz 1, 1030, Vienna, Austria; Centre for Microbiology and Environmental Systems Science, Department for Microbiology and Ecosystem Science, University of Vienna, Djerassiplatz 1, 1030, Vienna, Austria; Department for Soil Health and Plant Nutrition, Austrian Agency for Health and Food Safety (AGES), Spargelfeldstraße 191, 1220 Vienna, Austria; Department for Soil Health and Plant Nutrition, Austrian Agency for Health and Food Safety (AGES), Spargelfeldstraße 191, 1220 Vienna, Austria; Centre for Microbiology and Environmental Systems Science, Department for Microbiology and Ecosystem Science, University of Vienna, Djerassiplatz 1, 1030, Vienna, Austria; Joint Microbiome Facility of the Medical University of Vienna and the University of Vienna, Djerassiplatz 1, 1030, Vienna, Austria; Environment and Climate Hub, University of Vienna, Augasse 2/6, 1090 Vienna, Austria; Centre for Microbiology and Environmental Systems Science, Department for Microbiology and Ecosystem Science, University of Vienna, Djerassiplatz 1, 1030, Vienna, Austria; Environment and Climate Hub, University of Vienna, Augasse 2/6, 1090 Vienna, Austria; Centre for Microbiology and Environmental Systems Science, Department for Microbiology and Ecosystem Science, University of Vienna, Djerassiplatz 1, 1030, Vienna, Austria

**Keywords:** agricultural soils, ammonia oxidation, biological nitrification inhibitors, EC_50_, pH

## Abstract

Up to 70% of the nitrogen (N) fertilizer applied to agricultural soils is lost through microbially mediated processes, such as nitrification. This can be counteracted by synthetic and biological compounds that inhibit nitrification. However, for many biological nitrification inhibitors (BNIs), the interaction with soil properties, nitrifier specificity, and effective concentrations are unclear. Here, we investigated three synthetic nitrification inhibitors (SNIs) (DCD, DMPP, and nitrapyrin) and three BNIs [methyl 3(4-hydroxyphenyl) propionate (MHPP), methyl 3(4-hydroxyphenyl) acrylate (MHPA), and limonene] in two agricultural soils differing in pH and nitrifier communities. The efficacies of SNIs and BNIs were resilient to short-term pH changes in the neutral pH soil, whereas the efficacy of some BNIs increased by neutralizing the alkaline soil. Among the BNIs, MHPA showed the highest inhibition and was, together with MHPP, identified as a putative AOB/comammox-selective inhibitor. Additionally, MHPA and limonene effectively inhibited nitrification at concentrations comparable to those used for DCD. Moreover, we identified the effective concentrations at which 50% and 80% of inhibition is observed (EC_50_ and EC_80_) for the BNIs, and similar EC_80_ values were observed in both soils. Overall, our results show that these BNIs could potentially serve as effective alternatives to SNIs currently used.

## Teaser

Characterization of synthetic and biological nitrification inhibitors response in soil pH manipulations and effective concentrations.

## Introduction

In agricultural soils, nitrogen (N) often originates from the application of ammonium-based fertilizers, with ~115 Mt yr^−1^ of fertilizer applied globally (FAO 2019). However, modern fertilization practices are highly inefficient and between 50% and 70% of the applied N is lost from agricultural systems (Subbarao et al. 2015, Coskun et al. 2017). It is estimated that about 50% of the global anthropogenic nitrous oxide (N_2_O) emissions originate from fertilized agricultural soils and that by 2030 agricultural N_2_O emissions are projected to further increase another 15% (Tian et al. 2020, IPCC 2022). Continuing to feed the world’s growing population while reducing N losses from agriculture will, therefore only be possible with more efficient fertilization strategies.

A large fraction of N fertilizer applied to soils is lost through microbially mediated nitrification, a process in which ammonia (NH_3_) is oxidized to nitrite (NO_2_^−^) and subsequently to nitrate (NO_3_^−^). This process is mediated by three functional groups of microorganisms: ammonia-oxidizing archaea and bacteria (AOA and AOB), which oxidize NH_3_ to NO_2_^−^, nitrite-oxidizing bacteria (NOB), which oxidize NO_2_^−^ to NO_3_^−^, and complete ammonia-oxidizing (comammox) bacteria, which perform the complete oxidation of NH_3_ to NO_3_^−^ (Daims et al. 2015, van Kessel et al. 2015). As a result of nitrification, N is lost through leaching of highly mobile NO_2_^−^ and NO_3_^−^, or is emitted as N_2_O due to nitrifier denitrification, incomplete hydroxylamine oxidation and abiotic reactions between nitrification products (Giguere et al. 2017, Hink et al. 2018, Prosser et al. 2020, Hu et al. 2022). As ~90% of N fertilizer in soils is nitrified (Subbarao et al. 2013), nitrification plays a central role in agricultural N use management.

One approach to counteract N loss through nitrification in agricultural soils is the application of synthetic nitrification inhibitors (SNIs) together with N fertilizers (Huber et al. 1977, Slangen and Kerkhoff 1984, Ruser and Schulz 2015). Currently, the most widely used SNIs are dicyandiamide (DCD), 3,4–dimethylpyrazole phosphate (DMPP), and nitrapyrin (2-chloro-6-(trichloromethyl)-pyridine), which have been shown to effectively increase soil N retention while reducing fertilizer N losses (Zerulla et al. 2001, Subbarao et al. 2006, Zhou et al. 2020). Recently, plant-derived compounds that suppress soil nitrification, known as biological nitrification inhibitors (BNIs), have increasingly been considered as natural and inexpensive alternatives to SNIs (Subbarao et al. 2012, 2015, Coskun et al. 2017). Compounds isolated from root exudates, plant tissues, or plant residues, have previously been shown to have nitrification inhibition activity, including methyl 3-(4-hydroxyphenyl) propionate (MHPP) (Zakir et al. 2008), methyl 3-(4-hydroxyphenyl) acrylate (MHPA), also known as methyl-*p*-coumarate (Gopalakrishnan et al. 2007), and limonene (White 1991). However, conflicting results have been obtained when testing the efficacy of these and other BNIs on agricultural soils due to the influence of widely varying soil properties, the lack of robust estimates of effective BNI concentrations, and the variable responses of the different naturally occurring soil nitrifier communities.

In soils, factors such as pH, temperature, organic matter content, and the complex interactions among them, influence not only nitrifying communities and nitrification rates, but also the efficacy of nitrification inhibitors (NIs; Keeney 1980, Subbarao et al. 2015). In particular, soil pH controls many soil biotic and abiotic processes, such as the deprotonation of ammonium (NH_4_^+^) to NH_3_, which directly affects the activity of different soil ammonia oxidizers due to their different affinities for NH_3_ (Kits et al. 2017, Jung et al. 2022). In addition to this niche partitioning of ammonia oxidizers, soil pH also affects the sorption of NIs to soil particles, minerals, and soil organic matter; e.g. higher sorption of SNIs has been observed in alkaline soils (ASs) than in acidic soils (Guardia et al. 2018).

Inhibitor concentration is known to influence the efficacy of NIs (Nardi et al. 2020). In pure cultures of soil ammonia oxidizers, activity decreased linearly with BNI concentration (Kaur-Bhambra et al. 2022), suggesting that higher efficacies are achieved at higher BNI concentrations. Previous studies have shown the inhibitory effect of BNIs on soil nitrification (Zhang et al. 2015, Lu et al. 2019, Ma et al. 2021, Lan et al. 2022). However, different nitrification inhibition results were observed when using the same BNI concentration across different soils (Wang et al. 2021). Therefore, elucidating the factors that influence the efficacy of BNIs is an important first step in determining their potential efficiency in agricultural soils and whether they are a suitable natural alternative to SNIs in counteracting N fertilizer losses.

The objective of this study was to evaluate the efficacy of three SNIs (DCD, DMPP, and nitrapyrin) and three BNIs (MHPP, MHPA, and limonene) in two contrasting agricultural soils differing in pH and nitrifier communities. Moreover, for the three BNIs we determined the EC_50_ (effective concentration at which 50% of inhibition is observed) and EC_80_ in both soils. We hypothesized that soil pH has a strong direct influence on inhibitor efficacy by influencing soil sorption and physicochemical properties, but also indirectly, by driving differences in nitrifier abundance and diversity. We further hypothesized that BNI efficacy is concentration dependent, and that the EC_50/80_ of the tested BNIs differ between soils. While previous studies have assessed the efficacy of NIs in different soil types, this study examines the direct effect of short-term soil pH perturbations and inhibitor concentration on the efficacy of NIs.

## Materials and methods

### Site description and soil sampling

Samples were collected in spring 2022 from two agricultural soils located in Lower Austria, in the Marchfeld region (48°12′57.2″N 16°37′06.1″E) and in Alpenvorland (48°07′31.7″N 15°09′13.4″E). Both sites are part of long-term field experiments managed by the Austrian Agency for Health and Food Safety (AGES) (Lehtinen et al. 2014, Spiegel et al. 2018). The soil at the Marchfeld site is classified as Calcaric Phaeozem (sandy loam: 30.3% sand, 45.6% silt, and 24.2% clay), while the Alpenvorland site soil is a Gleyic Luvisol (loamy silt: 9.5% sand, 71.2% silt, and 19.4% clay). The two sites greatly differ in their soil pH: Marchfeld is an AS with a pH in water of 8.50 ± 0.02, while Alpenvorland is a slightly acidic or circumneutral soil (CS) with a pH of 6.12 ± 0.09. Over the last 40 years, both soils received 120 kg N ha^−1^ yr ^−1^, 75 kg P_2_O_5_ ha^−1^ yr ^−1^, and the crop rotation system consisted of 53%–55% cereals and 45%–47% root crops (Lehtinen et al. 2014, Spiegel et al. 2018). At each site, soil from the top 10 cm was collected from four field replicate plots, and each sample consisted of four soil cores. Samples were transported to the laboratory and sieved (2 mm mesh size). Soil for molecular analyses was stored immediately at −80°C, while soil for microcosm incubations was stored at 4°C.

### Soil characterization

Soil water content was determined gravimetrically by drying 5 g of soil at 60°C for 48 h. Dried and ground samples were analyzed for total carbon (C) and total N with an EA-IRMS (EA 1110, CE Instruments, Italy, coupled to a Finnigan MAT Delta Plus IRMS; Thermo Fisher Scientific, MA, USA). Soil pH was measured in water using standard pH electrodes (1:5 ratio w/v). The content of CaCO_3_, EDTA-extractable iron, manganese, copper, zinc, and CEC were determined by the Austrian Agency for Health and Food Safety (AGES) following the standard protocols ÖNORM L1084, L1098, and L1086-1. Microbial biomass C and N (C_mic_ and N_mic_) were determined by chloroform fumigation extraction and corrected for extraction efficiency using a factor of 0.45 (Vance et al. 1987, Jenkinson et al. 2004). Fumigated and nonfumigated soil samples were extracted with 1 M KCl (2 g soil in 15 ml KCl) and analyzed for dissolved organic carbon (DOC) and total dissolved N (TDN) content on a TOC/TN Analyzer (TOC-V CPH E200V/TNM-122 V; Shimadzu, Austria). A modified photometric indophenol reaction method was used to determine NH_4_^+^ in the KCl extracts (Kandeler and Gerber 1988), and the acidic VCl_3_/Griess reaction was used to determine NO_3_^−^ and NO_2_^−^ (Griess-Romijn van Eck 1966, Miranda et al. 2001).

### Microbial and nitrifier community characterization

DNA was extracted with the DNeasy PowerSoil Pro kit (Qiagen, Hilden, Germany) according to the manufacturer’s instructions. DNA concentrations were quantified fluorometrically with the Qubit dsDNA BR Assay Kit using an Invitrogen Qubit 4.0 Fluorometer (Thermo Fisher Scientific). AOA, AOB, and comammox ammonia monooxygenase subunit A (*amoA*) genes, which encode for the alpha subunit of the ammonia monooxygenase (AMO), were quantified by qPCR using SYBR Green Supermix in a CFX384 touch real-time PCR detection system (BioRad, USA). The 104F/616R (Alves et al. 2013), 1F/2R (Rotthauwe et al. 1997), and comaB_244F/comaB_659R (Pjevac et al. 2017) primers were used to quantify AOA, AOB, and comammox clade B *amoA* genes, respectively. Commamox clade A (comaA_244F/ comaA_659R) (Pjevac et al. 2017) *amoA* genes were not detected in any samples. Standard curves for each gene were generated from serial dilutions of 10^7^–10^1^ gene copies µl^−1^ of linearized plasmids with insertions of the target genes. All gene quantifications were performed in a 20-µl final reaction volume with triplicates of samples and standards. Further details on reagents and qPCR conditions are provided in Supplementary Table 1.

The *amoA* and 16S rRNA gene amplification and sequencing was carried out at the Joint Microbiome Facility of the Medical University of Vienna and the University of Vienna (JMF) under project IDs JMF-2110–10 and JMF-2206–04. For *amoA* gene sequencing, the primers described above for qPCR were used. The 515F/806R primers were used for 16S rRNA gene sequencing (Apprill et al. 2015, Parada et al. 2016). Target gene amplification and sample barcoding were performed with a two-step PCR protocol using the aforementioned primer pairs modified with linker sequences, as described in Pjevac et al. (2021). Sequencing was performed on the Illumina MiSeq platform, using the 600-cycle v3 chemistry (2 × 300 bp paired-end reads). Raw sequencing data were processed using FASTQ workflow (Basespace, Illumina) with default settings, and amplicon sequence variants (ASV) were inferred using the DADA2 pipeline (Callahan et al. 2016) using the recommended workflow. Taxonomy was assigned using the SILVA database SSU Ref NR 99 release 138.1 (Quast et al. 2013) for prokaryotic 16S rRNA gene amplicons. Custom databases for AOA, AOB, and comammox clade B *amoA* gene amplicons were created by retrieving all AMO entries for archaea, bacteria, and comammox clade B from NCBI GenBank. All generated amplicon sequence data were deposited at the NCBI Sequence Read Archive (SRA) under accession number PRJNA1031540.

### Soil net nitrification potential and AOA contribution

Soil slurry incubations were performed to evaluate the contributions of AOA and AOB/comammox to the net nitrification potential of both soils. For the assays, fresh soil (4 g) and 30 ml of MilliQ water were weighed in 125 ml Wheaton bottles, and NH_4_Cl was added to a final concentration of 1 mM. All bottles were sealed gas tight with 33 mm interlock butyl septa. To differentiate between the contribution of AOA and AOB/comammox to soil nitrification the AOB and comammox specific inhibitors 1-octyne (4 µM) and allyl-2-thiourea (ATU, 100 µM) (Sigma-Aldrich, St. Louis, MO, USA) treatments were included (Taylor et al. 2013, 2015). The negative control, in which nitrification activity was completely inhibited, received 1 mM phenylacetylene (McCarty and Bremner 1986). Sealed bottles were shaken at 180 r/m at 23°C for 72 h. Each treatment included four replicate soil slurries and 1 ml subsamples were collected with a 1-ml syringe through the butyl septa daily during the incubation period to measure NO_3_^−^ accumulation as described above. NO_2_^−^ accumulation was also assessed but was below the limit of quantification of 15 µM in all samples at all timepoints. Soil net nitrification potential (µg N g^−1^ soil day^−1^) was calculated by subtracting the initial NO_3_^−^ concentration from the accumulated NO_3_^−^ concentration, normalized to soil weight and incubation time.

### Short-term soil pH perturbations

To evaluate the effect of soil pH on the efficacy of SNIs and BNIs further soil slurry incubations were conducted as described above. The treatments consisted of modifying the pH of the soil slurries before NH_4_Cl and inhibitor addition. Soil slurries with the AS (native pH 8.50) were adjusted to pH 7.18 and 6.15 using 0.3 ml and 2.5 ml of 0.5 M HCl, respectively. Similarly, the pH of the CS slurries (native pH 6.12) was adjusted to pH 7.60 and 8.50 by adding 80 µl and 200 µl of 0.5 M NaOH, respectively. After the initial pH adjustment, slurries were left to stabilize for 24 h at 23°C, shaking at 180 r/m, before the addition of 1 mM NH_4_Cl and each of the inhibitors. The pH was measured periodically to confirm that the adjusted pH was stable throughout the experiment.

Nitrapyrin, DCD (Sigma-Aldrich), and DMPP (Cayman Chemical, Michigan, USA) were applied at rates previously used in agricultural soils (Lu et al. 2019, Dawar et al. 2021, Lan et al. 2022). Specifically, 3.5 µM nitrapyrin (equivalent to 24 µg g^−1^ soil) dissolved in 99.5% DMSO (Lactan Chemikalien & Laborgeräte GmbH, Graz, Austria) (0.035% v/v DMSO final concentration in the soil slurries), 100 µM DCD (equivalent to 252 µg g^−1^ soil) and 10 µM DMPP (equivalent to 58 µg g^−1^ soil) were added to the soil slurries. A DMSO control (0.035% v/v) was also included. As 1000 µg g^−1^ soil is the highest concentration of BNIs previously tested in different soil types (Wang et al. 2021), MHPP, MHPA, and limonene were tested at a concentration of 200 µM (equivalent to 1081.12, 1069.08, and 817.44 µg g^−1^ soil, respectively). In addition to the SNIs and BNIs, octyne, ATU, and phenylacetylene controls were included as described above. All treatments were performed in triplicates and incubated under the conditions described above. Previous experiments with the AS showed higher nitrification activity than the CS, so the incubation period for the AS was set to 48 h, while the incubations with the CS were carried out for 72 h. Subsamples of 1 ml were taken daily for NO_3_^−^ accumulation measurements to determine net nitrification potential as described above. The percentage of nitrification inhibition caused by each inhibitor was calculated using the following equation:


\begin{eqnarray*}
\textit{Nitrification}\,\,\textit{inhibition}\,\,\left( \% \right) = \,\,100\% - \left( {\frac{{T \times 100\% }}{{C\,\, + \,\,N}}} \right)
\end{eqnarray*}

where *T* is the NO_3_^−^ concentration (µg N g^−1^ dry soil) produced in the NI treatment, *C* is the NO_3_^−^ per gram of soil produced in the positive control (i.e. no inhibitor added) and *N* is the NO_3_^−^ consumption that occurred in the negative control (i.e. phenylacetylene addition).

### Net carbon dioxide production

Additionally, to assess the effect of the BNIs on the heterotrophic microbial community respiration, net carbon dioxide (CO_2_) production was quantified. Ambient and headspace gas samples were taken to determine initial (0 h) and final (72 h) CO_2_ concentrations in the treatments with and without BNI addition. CO_2_ concentrations were determined using an infrared gas analyzer (EGM-4 Environmental gas analyzer for CO_2_, PP Systems; Hertfordshire, UK). Net CO_2_ production in each treatment was calculated by subtracting the initial from the final CO_2_ concentration.

### EC_50_ and EC_80_ determination

To determine the EC_50_ and EC_80_ values of the three BNIs used in this study (MHPP, MHPA, and limonene) (Sigma-Aldrich), similar net nitrification potential incubations were performed as described above. For each BNI, up to six concentrations ranging from 50 µM to 1.5 mM were tested in each soil. All BNIs were dissolved in DMSO at concentrations ranging from 0.004% to 0.2% v/v, and a DMSO control (0.25% v/v) without inhibitors was also included. Moreover, octyne and phenylacetylene controls, as well as a positive control without inhibitors were included. The percentage of nitrification inhibition for each BNI concentration was calculated as described above. The EC_50_ and EC_80_ values for MHPP, MHPA, and limonene, were calculated by fitting a dose–response model (Motulsky and Christopoulos 2003) to the percentage of nitrification inhibition at each concentration using the equation:


\begin{eqnarray*}
y = Min + \,\,\frac{{Max - Min}}{{1 + {{\left( {\frac{{\frac{{E{C_F}}}{{{{\left( {\frac{F}{{100 - F}}} \right)}^{\frac{1}{h}}}}}}}{x}} \right)}^h}}}
\end{eqnarray*}

where *y* is the percentage of nitrification inhibition, *x* is the concentration of inhibitor (µM), *h* is the Hill coefficient, *F* is the target percentage of inhibition (i.e. 50% or 80%), and *EC_F_* is the effective concentration at which 50% or 80% of inhibition is observed. The model was constrained to two parameters, setting the minimum (*Min*) and maximum (*Max*) effect of inhibition to 0% and 100%, respectively. Additionally, the target percentage of inhibition (*F*) was set to either 50 for EC_50_ or to 80 for EC_80_ calculations. These constraints simplified the equation to:


\begin{eqnarray*}
y = \frac{{100}}{{1 + {{\left( {\frac{{\frac{{E{C_F}}}{{{{\left( {\frac{F}{{100 - F}}} \right)}^{\frac{1}{h}}}}}}}{x}} \right)}^h}}}
\end{eqnarray*}

Nonlinear least squares regression (Kemmer and Keller 2010) was used to estimate the EC_50_ and EC_80_ values as well as the Hill coefficient, along with their respective standard errors.

### Statistical analysis

All statistical analyses were performed in R version 4.3.1 (R Core Team 2023) using Rstudio version 2023.6.0.421 (Posit Team 2023). Differences in soil parameters between sites were tested using a *t*-test (for Mn, Cu, Zn, DOC, soil C:N, C_mic_, N_mic_, microbial C:N, ammonium, and nitrate) or a Wilcoxon signed-rank test (for pH, % CaCO_3_, Fe, CEC, total C, and total N). For the differences in ammonia oxidizer abundances, a generalized least squares model was run using the *gls* function of the *nlme* 3.1–162 package (Pinheiro et al. 2023). Here, ‘site’ and ‘type of ammonia oxidizer’ were used as fixed factors and the abundance of each gene as the response variable. A two-way ANOVA was used to test for differences in the total net nitrification potential and AOA activity between soils. *T*-tests were conducted to compare the efficacy of each NI among sites and a one-way ANOVA was used to test whether the inhibitor efficacy depended on adjusted soil pHs. For all tests, a *P-*value < .05 was considered to mark significant differences between sites or inhibitor treatments, unless otherwise noted.

Sequence data analyses were performed using the *phyloseq* 1.44.0 (McMurdie and Holmes 2013), and *vegan* 2.6–4 packages (Oksanen et al. 2022). For all genes, a canonical correspondence analysis on a Bray–Curtis dissimilarity matrix was performed using the *ordinate* function from the *phyloseq* package. The significance of the environmental variables was assessed with the *envifit* function of the *vegan* package and only the variables with a *P* < .01 were chosen for the canonical correspondence analysis (Supplementary Table 2). Alpha-diversity was determined on a dataset rarefied to 6126, 3189, 905, and 2688 reads sequencing depth for the 16S rRNA, AOA, AOB, and comammox clade B *amoA* gene datasets, respectively. To test for the differences in the 16S rRNA and *amoA* alpha diversity between sites, a *t*-test or a Wilcoxon signed-rank test were performed. To test for significant differences between the taxa with mean relative abundance ≥ 2% for the 16S rRNA, and ≥ 5% for the *amoA* gene amplicon datasets, the ANCOMBC 2.2.0 (Lin and Peddada 2020) package was used.

## Results

### Soil physicochemical properties of the studied sites

Two distinct agricultural soils with differing pH were selected for this study, an AS (AS, pH 8.50 ± 0.02) and a CS (pH 6.12 ± 0.09) (Table 1). While many soil properties significantly differed between the soils, the TDN and EDTA-extractable zinc did not. Besides a high pH, the AS is characterized by a significantly higher CaCO_3_ content, CEC, EDTA-extractable (i.e. plant available) copper concentration, and nitrate concentration. In addition, the AS also has a significantly higher total C, total N, soil C:N ratio, DOC, microbial C, and microbial N. The CS, in contrast, has significantly higher ammonium, and EDTA-extractable iron and manganese concentrations (Table 1).

**Table 1.** Soil parameters of the AS and the CS. CEC: cation exchange capacity; soil C:N: molar based soil C:N ratio; DOC: dissolved organic carbon; TDN: total dissolved nitrogen; C_mic_: microbial biomass C; N_mic_: microbial biomass N; and microbial C:N: molar based microbial C:N ratio. Samples collected in spring 2022 (*n* = 4, SE: standard error).

	AS		CS		*P*
	Mean	SE	Mean	SE	
pH	8.50	± 0.02	6.12	± 0.09	0.028
% CaCO_3_	12.0	± 0.56	0	± 0.0	0.021
Iron (mg kg^−1^)	65.5	± 2.75	480.5	± 17.69	0.028
Manganese (mg kg^−1^)	149.8	± 22.75	375.5	± 7.50	< 0.001
Copper (mg kg^−1^)	6.0	± 0.31	4.7	± 0.04	0.026
Zinc (mg kg^−1^)	2.6	± 0.25	2.4	± 0.09	0.534
CEC (cmolc kg^−1^)	27	± 0.41	8.8	± 0.36	0.028
Total C (mg g^−1^ dw soil)	23.23	± 1.07	8.40	± 0.14	0.028
Total N (mg g^−1^ dw soil)	2.35	± 0.03	1.08	± 0.01	0.028
Soil C:N	11.54	± 0.44	9.06	± 0.09	0.009
DOC (µg C g^−1^ dw soil)	68.88	± 2.41	10.21	± 2.29	0.001
TDN (µg N g^−1^ dw soil)	20.26	± 0.80	14.96	± 3.03	0.142
C_mic_ (µg C g^−1^ dw soil)	179.33	± 9.35	133.91	± 4.70	0.005
N_mic_ (µg N g^−1^ dw soil)	41.83	± 3.58	17.09	± 2.40	0.003
Microbial C:N	5.13	± 0.54	9.53	± 1.65	0.034
NH_4_^+^ (µg N g^−1^ dw soil)	3.26	± 0.87	10.12	± 2.32	0.032
NO_3_^−^ (µg N g^−1^ dw soil)	8.49	± 0.24	3.65	± 0.35	< 0.001

### Total microbial community composition

**Figure 1.**
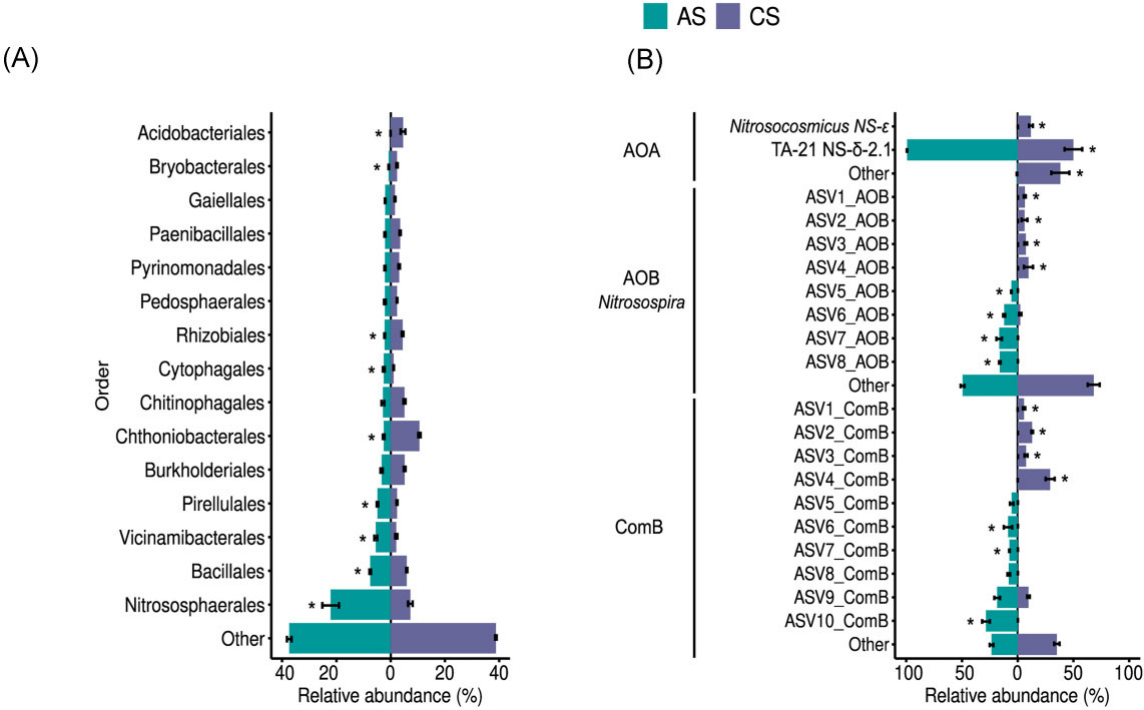
Soil microbial community composition in the AS and the CS. (A) 16S rRNA gene-based microbial community composition depicting the top taxa with ≥2% mean relative abundance at each site (B) *amoA* microbial community composition depicting the top *amoA* taxa with ≥5% mean relative abundance. Taxa with mean relative abundance below 2% (for 16S rRNA gene) or 5% (for *amoA*) are clustered as ‘other’. Samples collected in spring 2022 (*n* = 4, error bars represent standard error) (*P < .001). Absolute abundances of the *amoA* genes are shown in Supplementary Fig. 3.

Microbial community structure, based on 16S rRNA gene amplicon sequencing, significantly differed between the AS and CS in the most abundant orders (only orders with ≥ 2% mean relative abundance at each site were compared; Fig. 1A). In both soils, the AOA order Nitrososphaerales was among the most abundant microbial orders but accounted for a significantly higher relative abundance in the AS than in the CS (*P* < .0001). In the AS, the Nitrososphaerales comprised 22 ± 3.05% of the 16S rRNA gene-based community, while in the CS they represented only 7.2 ± 0.84% (Fig. 1A). Other putative nitrifiers within the genera *Nitrospira* (1.4 ± 0.25% in the AS and 0.99 ± 0.11% in the CS) and *Nitrosospira* (0.27 ± 0.03% in the AS and 0.28 ± 0.02% in the CS) were also detected in both soils, but at low relative abundances (<2%).

The diversity of the total microbial community was significantly higher in the CS, while the richness and observed diversity did not differ significantly between the two sites (Supplementary Fig. 1). Microbial community composition dissimilarities between the two investigated soils corresponded to differences in pH, CEC, and DOC, which were higher in the AS, as well as Fe and Mn, which were higher in the CS. Together, these factors explain over 62.1% of the community variance along the CCA1 (Supplementary Fig. 2).

### Ammonia oxidizer community and net nitrification potential

Analysis of the ammonia oxidizer community with *amoA* gene amplicon sequencing also showed significant differences between the AS and the CS. The AOA communities were dominated by ASVs affiliated with the genus TA-21 from the NS-δ-2.1 clade (Alves et al. 2018), representing 99.2 ± 0.2% of the AOA ASVs in the AS and 49.9 ± 7.8% in the CS (Fig. 1B, Supplementary Fig. 3). Additional AOA *amoA* ASVs, belonging to the NS-ε clade (11.79 ± 1.7%) were only present in the CS. Members of the well-known terrestrial AOB genus *Nitrosospira* were dominant in both soils, but distinct ASVs were detected between each soil. Similarly, distinct comammox clade B ASVs were detected between soils (Fig. 1B). Both richness and evenness of the AOB and comammox clade B communities significantly differed between the AS and CS, while not significant differences were observed in the AOA community (Supplementary Fig. 4). Much like with the 16S rRNA total microbial community, pH and DOC were the strongest drivers of *amoA* community dissimilarities (Supplementary Fig. 5).

**Figure 2.**
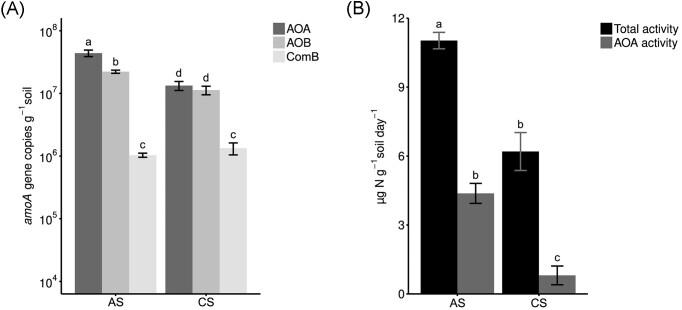
Abundance of ammonia oxidizers, total net nitrification potential activity, and AOA contribution to the net nitrification potential. (A) Absolute abundance of ammonia oxidizers in the AS and the CS. (B) Total net nitrification potential activity and AOA contribution to the net nitrification potential. Lower case letters depict significant differences within and between soils. Samples collected in spring 2022. Error bars represent the standard error in each case (*n* = 4, *P* < .05).

The quantification of the *amoA* genes showed significant differences in the total *amoA* gene abundance between sites. Overall, the AS harboured a higher number of *amoA* genes than the CS (6.7 × 10^7^ ± 2.5 × 10^6^  *amoA* gene copies g^−1^ soil and 2.5 × 10^7^ ± 1.8 × 10^6^  *amoA* gene copies g^−1^ soil, respectively; *P* = < .01). In fact, the AS showed significantly higher abundances of both AOA and AOB; however, both sites contained similar comammox clade B abundances (Fig. 2A). No comammox clade A *amoA* genes were detected in either soil. The AS soil was dominated by AOA, containing 4.3 × 10^7^ ± 5.2 × 10^6^  *amoA* AOA gene copies g^−1^ soil. In addition, 2.2 × 10^7^ ± 1.2 × 10^6^  *amoA* gene copies g^−1^ soil and 1.0 × 10^6^ ± 7.7 × 10^4^  *amoA* gene copies g^−1^ soil of the AOB, and comammox clade B communities were detected, respectively (Fig. 2A). In contrast, the CS had similar AOA and AOB abundances (1.3 × 10^7^ ± 2.1 × 10^6^  *amoA* gene copies g^−1^ soil and 1.1 × 10^7^ ± 1.7 × 10^6^  *amoA* gene copies g^−1^ soil, respectively) and less comammox clade B bacteria (1.3 × 10^6^ ± 2.8 × 10^5^  *amoA* gene copies g^−1^ soil) (Fig. 2A).

The total net nitrification potential and octyne resistant (i.e. AOA contribution) activity fraction were determined in both soils. Total net nitrification potential was 2-fold and 10-fold higher than octyne resistant activity in AS and CS, respectively (Fig. 2B). In the AS the octyne resistant fraction contributed about ∼35% of the total activity, compared to less than 7% in the CS (Fig. 2B). Overall, the AS had higher total net nitrification potential and octyne resistant activity, along with a larger total ammonia oxidizer and AOA abundances.

### Differences in NI efficacy between soils

**Figure 3.**
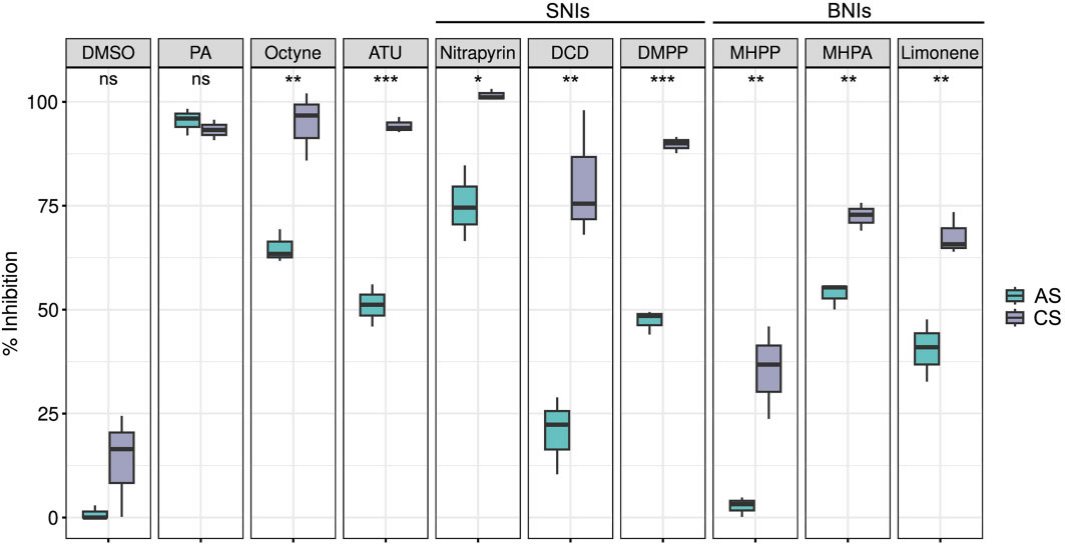
Efficacy of SNIs and BNIs in the AS and CS. DMSO: dimethyl sulfoxide control; PA: phenylacetelyne. 1 mM PA, 4 µM Octyne, 100 µM ATU, 3.5 µM Nitrapyrin, 100 µM DCD, 10 µM DMPP, 200 µM MHPP, 200 µM MHPA, and 200 µM limonene. Samples collected in spring 2022. The median is depicted as the middle hinge in the boxplots. Upper and lower hinges represent the first and third quartile. The length of the whispers is determined by the largest and the smallest value in the dataset that are within 1.5 times the interquartile range. (*n* = 3, *P < .05, **P < .01, and ***P < .001, ns: not significant).

Due to the low solubility of nitrapyrin, MHPP, and MHPA in water, these NIs were dissolved in DMSO for application and therefore the effect of DMSO (0.035% v/v) alone on the net nitrification potential was also assessed in both soils. DMSO had no significant effect on the net nitrification potential (Supplementary Fig. 6, *P* = .106 in the AS and *P* = 0.747 in the CS). In addition, phenylacetylene (1 mM) was used as a total net NI and caused 95.4 ± 1.8% and 93.2 ± 1.4% inhibition in the AS and CS, respectively (Fig. 3). The AOB and comammox-selective inhibitors, octyne and ATU, were used to differentiate between bacterial and archaeal ammonia oxidizer contribution to net nitrification activity in both soils. As expected, octyne and ATU showed similar nitrification inhibition patterns across both soils. In the AS, octyne (4 µM), and ATU (100 µM) inhibited 65 ± 2.3% and 51 ± 2.9% of the net nitrification potential, respectively, indicating that the remaining activity was AOA driven. In contrast, both compounds inhibited ∼94% of the net nitrification potential in the CS, highlighting that the ammonia oxidation activity in this soil was almost exclusively AOB/comammox driven.

The efficacy of all tested SNIs and BNIs was significantly higher in the CS than in the AS (Fig. 3). Overall, the efficacy of SNIs showed greater variability in the AS with inhibition efficacies between 20% and 75%, while in the CS all SNIs were highly effective, inhibiting 80%–100% of nitrification. Among the three SNIs tested, nitrapyrin was the most effective inhibitor in both soils, with an average of 75 ± 5.2% net nitrification inhibition in the AS and 100 ± 0.7% in the CS. DMPP inhibited net nitrification potential by 47 ± 1.6% in the AS and 90 ± 1.1% in the CS. DCD was the least effective SNI tested in both soils, with 20 ± 5.4% and 80 ± 9% of inhibition in the AS and the CS, respectively (Fig. 3).

Among the three BNIs, MHPA had the highest inhibition efficacy in both soils with 54 ± 1.8% and 72 ± 1.9% of inhibition in the AS and CS, respectively. That was followed by limonene with 40 ± 4.3% of inhibition efficacy in the AS and 68 ± 2.9% in the CS. MHPP was the least effective BNI tested in both soils, inhibiting 35 ± 6.4% in the CS, and having almost no effect on the net nitrification potential in the AS with 2.7 ± 1.3% of inhibition (Fig. 3). Interestingly, all inhibitors (selective inhibitors, SNIs, and BNIs) had a significantly higher efficacy in the CS at pH 6.12 ± 0.09 than in the AS at pH 8.50 ± 0.02.

### NI efficacy is not exclusively driven by soil pH

**Figure 4.**
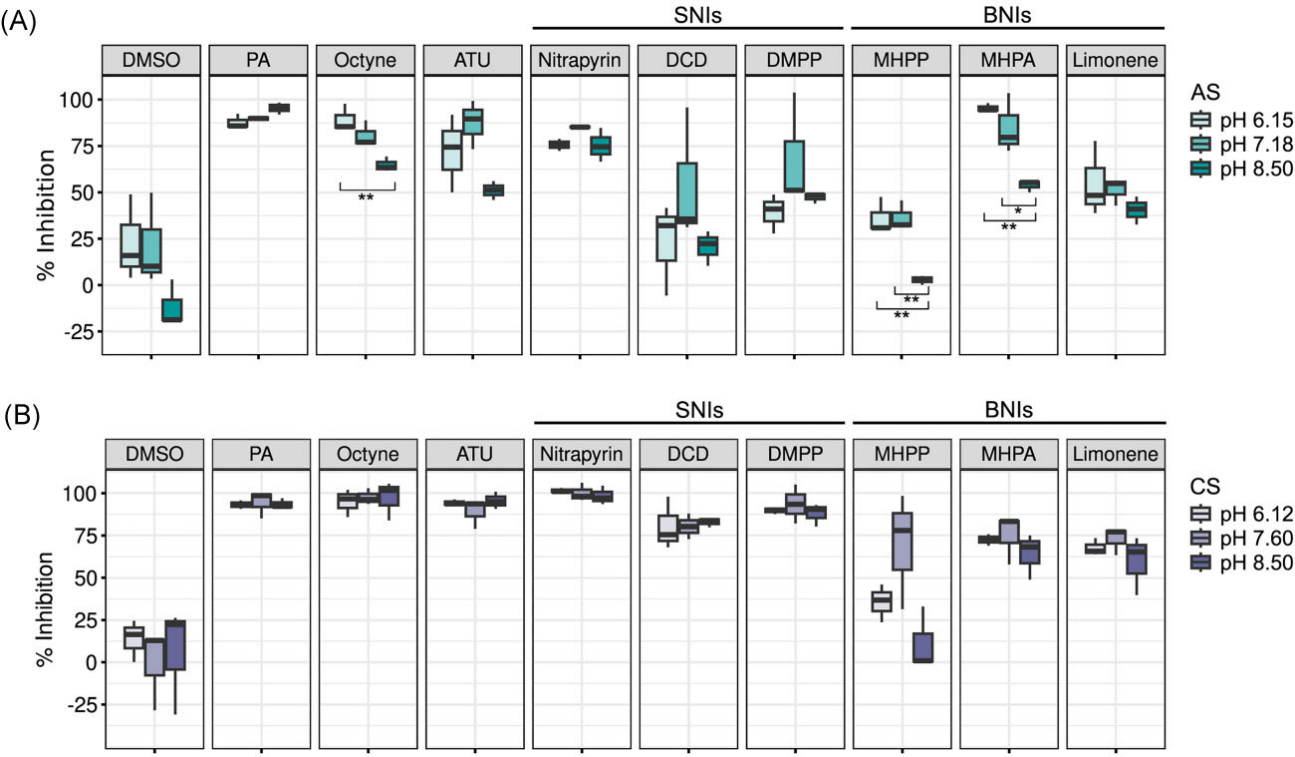
Effect of soil pH manipulation on the efficacy of SNIs and BNIs in the (A) AS (pH 8.50), (B) CS (pH 6.12) PA: 1 mM Phenylacetelyne, 4 µM Octyne, 100 µM ATU, 3.5 µM Nitrapyrin, 100 µM DCD, 10 µM DMPP, 200 µM MHPP, 200 µM MHPA, and 200 µM limonene. Samples collected in spring 2022. The median is depicted as the middle hinge in the boxplots. Upper and lower hinges represent the first and third quartile. The length of the whispers is determined by the largest and the smallest value in the dataset that are within 1.5 times the interquartile range. (*n* = 3, *P < .05, **P < .01).

As soil pH was significantly different between the two analysed soils and has previously been reported to affect NI efficacy (Keeney 1980, Bachtsevani et al. 2021, Yin et al. 2023), we hypothesized that the observed differences in the efficacy of the BNIs and SNIs are predominantly driven by soil pH. Therefore, short-term soil pH manipulations were performed for each soil and the efficacy of all NIs at the modified pHs was determined. Interestingly, pH modifications by almost one pH unit (from 8.5 to pH 7.18) significantly increased the net nitrification potential in the AS, while a decrease by a second pH unit (from 8.5 to pH 6.15) did not significantly impact net nitrification potential (Supplementary Fig. 7A). In contrast, pH modifications by one pH unit (from 6.15 to pH 7.6) did not significantly impact the overall net nitrification potential in the CS but pH modifications by a second pH unit (from 6.12 to pH 8.5) resulted in a significant decrease in the net nitrification potential (Supplementary Fig. 7B). Importantly, net nitrification was still observed in all pH modified soils (Supplementary Fig. 7). In each soil, the pH modifications did not have a significant effect on the percentage of inhibition in the phenylacetylene or DMSO controls (Fig. 4). For all the tested NIs, the percentage of inhibition at a given soil pH was determined relative to the respective net nitrification potential at that pH.

In the AS, when the pH was reduced from 8.5 to 6.15, the AOA-driven net nitrification potential decreased, as observed by a significant increase in the efficacy of octyne (24%, *P* = < .01, Fig. 4A). Similarly, the efficacy of MHPP and MHPA also significantly increased (34% and 42%, respectively) when the soil pH was reduced from 8.5 to 6.15 (Fig. 4A). Interestingly, the efficacy of octyne and MHPP (*r* = 0.80 *P* = < .01) as well as octyne and MHPA (*r* = 0.92, *P* = < .001) were positively correlated. While limonene followed a similar pattern where the highest efficacy was observed at pH 6.15, its efficacy across soil pHs did not significantly correlate with the efficacy of octyne (*r* = 0.32, *P* = .4, Supplementary Fig. 8). In contrast, the efficacy of the three SNIs tested was not significantly affected by the soil pH modifications in the AS. In the CS, raising the soil pH from 6.12 to 8.5 did not have a significant effect on the efficacies of any of the selective inhibitors, BNIs, or SNIs (Fig. 4B).

### BNI effective concentrations

**Figure 5.**
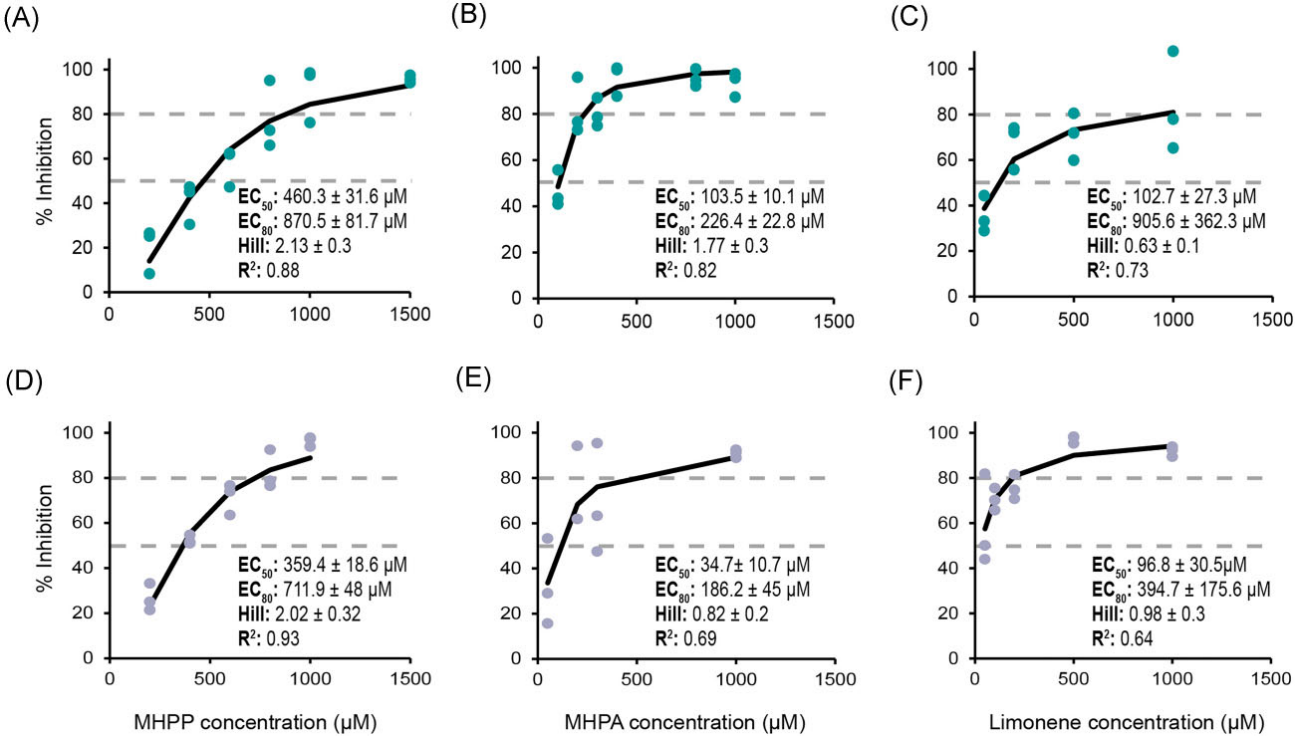
Log-logistic model fitting to estimate the EC_50_ and EC_80_ values of three BNIs in the AS and CS. (A) and (D) log-logistic model for MHPP, (B) and (E) for MHPA, and (C) and (F) for limonene. (A), (B), and (C) correspond to the inhibitors assessed in the AS, and (D), (E), and (F) in the CS. The corresponding EC_50_, EC_80_ values (dashed lines), and hill coeficient, with their respective standard errors, as well as the *R*^2^ are displayed for each BNI in each soil.

An important characterization of NIs is the concentration or field application rate necessary to effectively inhibit soil nitrification. Due to the differences in soil physicochemical properties, ammonia oxidizer community, and net nitrification activity between the AS and CS, we hypothesized that the EC_50_ and EC_80_ values of the three tested BNIs would differ significantly between the soils. This was the case for the EC_50_ values of MHPP (460.3 ± 31.6 µM in the AS and 359.4 ± 18.6 µM in the CS) and MHPA (103.5 ± 10.1 µM in the AS and 34.7 ± 10.7 µM in the CS), which both had significantly lower EC_50_ values (i.e. higher efficacy) in the CS (Fig. 5, Supplementary Table 3). However, the EC_50_ values of limonene were not significantly different between the AS and the CS. Among the three BNIs tested, MHPP again had the lowest efficacy (highest EC_50_ and EC_80_ values) in both soils. Notably, the EC_80_ values of all three tested BNIs were not significantly different between soils (Fig. 5, Supplementary Table 3).

## Discussion

### Efficacy of NIs in two contrasting agricultural soils

While variations in NI efficacy between soils have previously been reported (Lu et al. 2019, Zhou et al. 2020, Bachtsevani et al. 2021, Ma et al. 2021, Wang et al. 2021, Yin et al. 2023), the extent to which soil pH, soil physicochemical properties, and microbial community affect the efficacy of SNIs and BNIs remains largely unexplored. Consequently, in this study, the efficacy of three BNIs: MHPP, MHPA, and limonene, and three SNIs: nitrapyrin, DCD, and DMPP, in two contrasting agricultural soils were investigated. The efficacy of all the NIs tested here was higher in the CS (pH 6.12 ± 0.09) than in the AS (pH 8.50 ± 0.02) (Fig. 3). While the CS had similar abundances of AOA and AOB/comammox, the net nitrification potential was AOB/comammox driven with AOA activity contributing <7% (Fig. 2). In contrast, the abundance of AOA in the AS soil was higher than the abundances of AOB/comammox, and contributed ∼35% of the total net nitrification potential. Although the incubation conditions used in this study might have altered the proportion of AOB/comammox and AOA activities compared to the more competitive interactions observed in whole soil incubations (Prosser and Nicol 2012, Rütting et al. 2021). This trend of higher NI efficacy in more neutral soils, where net nitrification potential is AOB driven, compared to AOA dominated or more ASs has been observed previously (Guardia et al. 2018, Lu et al. 2019, Lei et al. 2022).

In our study, the SNI nitrapyrin was the most effective NI regardless of soil type even when applied at a low field application rate (3.5 µM, 0.35% of the N applied) (Fig. 3). This is in line with other studies comparing the efficacy of SNIs, where nitrapyrin is the most effective inhibitor regardless of soil type (Hayden et al. 2021, Lan et al. 2023), as long as it is applied at a sufficient rate (Zhou et al. 2020). In agreement with previous reports (Guardia et al. 2018, Zhou et al. 2020, Lan et al. 2023), DMPP was more effective than DCD, even though the applied DCD concentration was 10 times higher (10 µM and 100 µM, respectively).

Similar to the trend observed for the SNIs, the efficacy of all BNIs tested here was also higher in the CS than in the AS, which is in agreement with previous studies, which observed greater BNI inhibition efficacy in acidic soils compared to ASs (Nardi et al. 2013, Lu et al. 2019, Subbarao et al. 2021). Among the BNIs tested, MHPA had the highest efficacy in both soils followed by limonene (Fig. 3). Notably, this is the first study assessing the efficacy of MHPA and limonene in agricultural soils. MHPP, a commonly tested BNI, showed the lowest inhibition efficacy of all the BNIs and SNIs tested in both soils (Fig. 3). Although MHPP has shown higher efficacy compared to DCD in a soil of similar pH as the CS (Nardi et al. 2013), a lower efficacy of MHPP has also been reported previously in ASs (Lu et al. 2019, He et al. 2023). Together, our results support the majority of previous studies, which also report higher BNI efficacy in lower-pH soils (Lu et al. 2019, Subbarao et al. 2021).

Overall, the differences in NIs efficacy across soils observed here and in several other studies (Shi et al. 2016, Zhou et al. 2020, Lu et al. 2022, Lan et al. 2023), suggest that the efficacy of SNIs and BNIs in soils is affected by soil pH (Shi et al. 2016, Lu et al. 2022), the varying physicochemical properties of soils (Marsden et al. 2016, McGeough et al. 2016, Guardia et al. 2018) and the microbial community abundance and composition (Zhou et al. 2020, Bachtsevani et al. 2021).

### The role of pH on NI efficacy

Significant differences in NI efficacies between soils of differing pH were observed here and have been previously reported (Shi et al. 2016, Lu et al. 2019, Zhou et al. 2020, Bachtsevani et al. 2021). However, a recent study concluded that pH is not the main driving factor of the efficacy of the SNI, DMPP in acidic agricultural soils (Oliveira et al. 2022). Therefore, we conducted short-term soil pH manipulation experiments to determine if soil pH directly acts as the main driver of the differences observed in NI efficacy. Contrary to our hypothesis, the efficacy of most SNIs and BNIs was not significantly affected by short-term soil pH manipulations (Fig. 4). This suggests that soil pH alone is not the main driver of the differences observed here in NI efficacy between the AS and CS. Instead, other soil physicochemical properties such as organic matter, clay content, and the concentration of specific micronutrients may have a more direct short-term effect on the efficacy of NIs in soils.

### The role of soil physicochemical properties on NI efficacy

Previous studies have identified that sorption of NIs to organic matter can affect NI efficacy in soils (Marsden et al. 2016, Guardia et al. 2018). Under the incubation conditions used here, it is likely that the interactions of NIs with the dissolved organic matter are facilitated and therefore, a lower NI efficacy is expected in the soil with high organic content. The AS did not only have higher pH, but also higher total N, total C and CEC (Table 1), suggesting that the lower efficacy of all NIs tested may be due to higher sorption of the NIs to the soil matrix (Fig. 3). Previously, a strong correlation between NI half-life time and total N across nine different soil types was observed, where for instance, the half-life time of DCD was reduced in a soil with similar total N and CEC as the AS in our study (McGeough et al. 2016). Aside from organic matter, clay particles can also reduce the bioavailability of NIs through sorption. In our study, the clay contents of the AS and CS were comparable (24.2% in the AS and 19.4% in the CS). Among the NIs tested here, MHPP had the lowest efficacy in both soils (Fig. 3). This is in agreement with the low efficacy of MHPP, observed in a soil with similar clay content as that of the two soils tested here (Lu et al. 2019), and in contrast to the higher efficacy of MHPP in a soil with a very low clay content (2.1%) (Nardi et al. 2013). Altogether, these observations suggest that high organic matter, CEC, and clay content directly affects the efficacy of NIs by reducing their mobility, half-life time or bioavailability.

In addition, the availability of micronutrients, such as iron and copper, also influences ammonia oxidation (Reyes et al. 2020, Shafiee et al. 2021). While the exact mode of action of many NIs is unclear, it has been proposed that several NIs (e.g. DCD and DMPP) can act as copper chelators (Supplementary Table 4) suggesting their efficacy is affected by soil copper content. The significantly higher EDTA-extractable copper content in the AS (Table 1) is one possible explanation for the high net nitrification potential activity (Fig. 2B), as well as for the generally lower NI efficacy observed in the AS (Fig. 3). In fact, a negative correlation between copper content and nitrification inhibition by DCD has been previously reported (McGeough et al. 2016). Likewise, the low organic matter content, CEC, and copper content in the CS may have facilitated the higher inhibition efficacy observed for all NIs tested. However, the individual effects of soil organic matter, clay, and copper content on NI efficacy can not be determined from this study as all three soil properties covaried, were significantly higher in the AS soil, and likely have additive effects (Table 1).

### Role of the soil microbial community on NI efficacy

Both SNIs and BNIs showed higher efficacy in the CS, which had fewer ammonia oxidizers in total, a lower net nitrification rate, and the AOB/comammox communities contributed to >90% of the net nitrification potential (Figs 2 and 3). Regarding the role of ammonia oxidizer community abundance and diversity on NI efficacy in soils, a selective inhibition of AOB over AOA has been proposed (Zhou et al. 2020, Bachtsevani et al. 2021, Nair et al. 2021). Notably, despite the exceptionally high relative abundance of AOA (∼22%) and the 1.9-fold higher copy number of AOA amoA genes than AOB amoA genes in the AS, only 35% of total activity was octyne resistant (i.e. AOA driven). A recent meta-analysis on the effects of SNIs on the microbial community concluded that the application of DMPP, DCD, and nitrapyrin significantly reduced the gene and transcript abundances of AOB *amoA*, rather than AOA *amoA* (Yin et al. 2023). This selective effect of DMPP has also been observed when tested with ammonia-oxidizing pure cultures (Papadopoulou et al. 2020) and in soils where nitrification activity was predominantly AOB driven (Bachtsevani et al. 2021). The higher NI efficacy in the CS observed here (Fig. 3) may further indicate that AOB/comammox are more susceptible to SNIs and BNIs than AOA. However, the AS and CS harbour distinct AOB/comammox communities (Fig. 1B). These observed differences in nitrifying communities may be driven by long-term differences in pH, which taken together may also contribute to the differences in NI efficacy observed.

Interestingly, lowering the pH in the AS caused a significant increase in the efficacy of the selective inhibitor, octyne, suggesting that AOA activity decreased with decreasing pH (Fig. 4). Decreasing AOA activity at lower pH is in contrast to other studies which have observed that nitrification is most often AOA-driven in low-pH soils (Prosser and Nicol 2012, Zhang et al. 2012). The AOA community in the AS was dominated by the genus TA-21 from the NS-δ-2.1 clade (Fig. 1 and Supplementary Fig. 3). This AOA clade has been previously found in soils (Lee et al. 2023), but significant contributions to nitrification have not been reported previously and little is known about the pH preferences or physiology of AOA in the NS-δ-2.1 clade. However, under the incubation conditions used here, the genus TA-21 (NS-δ-2.1 clade) was seemingly responsible for the octyne-resistant fraction observed in the AS as it makes up 99.2 ± 0.2% of the AOA community in this soil (Fig. 1 and Supplementary Fig. 3).

Notably, in addition to octyne, MHPP and MHPA also displayed a seemingly AOB/comammox selective inhibition behaviour. This is illustrated by the strong positive correlation between their efficacy and the efficacy of octyne in the AS across the pHs tested (Supplementary Fig. 8). A similar indication that MHPP is a potential selective inhibitor of AOB/comammox was recently observed with ammonia-oxidizing pure cultures (Kaur-Bhambra et al. 2022), where double the inhibitor concentration was required to inhibit soil AOA cultures compared to soil AOB cultures. In contrast, direct evidence from ammonia-oxidizing pure cultures is required to confirm the selective inhibition potential of MHPA.

Aside from the different ammonia oxidizer communities, other members of the soil microbial community may also influence NI efficacy. Higher soil microbial biomass has been previously suggested as an indicator for higher microbial NI degradation (McGeough et al. 2016). Additionally, high CO_2_ respiration rates have been observed for the commonly used SNIs DMPP and DCD (Guardia et al. 2018). Therefore, it is expected that organic compounds such as BNIs will also be susceptible to microbial uptake, transformation, and degradation which may affect NI efficacy (Marsden et al. 2016). While the CS and the AS harbour distinct microbial communities, the AS has significantly higher microbial N and C biomass than the CS (Fig. 1 and Table 1), which is an additional explanation of the overall lower NI efficacy in the AS. In support of this hypothesis, significantly higher CO_2_ production was observed in the MHPP and MHPA treatments in the AS and the CS, compared to the control without BNI addition (Supplementary Fig. 9). Although the C added by the application of BNIs (200 µM equivalent to 0.18 mg C g^−1^ soil) represented only 0.8% and 2% of the total C content in the AS and the CS (Table 1), respectively, more C as CO_2_ was produced than what can be attributed to the BNIs alone. These observations suggest that the addition of BNIs might indirectly stimulate other soil microbial processes, which could subsequently affect the microbial uptake, transformation, degradation, and ultimately the efficacy of NIs in soils.

### Effective concentrations of BNIs in two contrasting agricultural soils

While there has been an increasing interest in the applicability of BNIs in agroecosystems (Lu et al. 2019, Wang et al. 2021, Lan et al. 2022, 2023), this study is the first to report the efficacy (EC_50_ and EC_80_ values) of three BNIs: MHPP, MHPA, and limonene in agricultural soils. All three BNIs tested here followed a log-logistic inhibition model (Fig. 5). Interestingly, while the EC_50_ values of MHPP and MHPA differed between soils, the EC_80_ values of the three BNIs were similar between the AS and the CS.

Previous reports of EC_50_ values for BNIs are scarce, yet the EC_50_ values for the three BNIs tested here are within similar ranges to the values previously reported. With an EC_50_ 34.7 ± 10.7 µM in the CS, the EC_50_ of MHPA is comparable to the value previously determined with the AOB, *Nitrosomonas europaea* (19.5 µM) (Gopalakrishnan et al. 2007). Similarly, the EC_50_ of limonene in the CS (96.8 ± 30.5 µM), is only 2.5-fold higher than the value previously reported for *N. europaea* (38 µM) (Ward et al. 1997). A more recent study reported EC_80_ values of MHPP in several pure AOA and AOB cultures, and ranged between 78.8 and 647.3 µM for soil AOA, and between 46.8 and 341.3 µM for soil AOBs (Kaur-Bhambra et al. 2022). While the EC_80_ values of MHPP in the CS (711.9 ± 48 µM) and the AS (870.5 ± 81.7 µM) are higher than the values reported for soil AOB cultures, our EC_80_ values are very similar to the highest effective concentration of MHPP for soil AOA cultures. Overall, higher EC_50_ and EC_80_ values for MHPA, MHPP, and limonene were observed in the AS and in the CS than in previous pure culture studies, which is likely a result of a more complex soil microbial community and interactions with soil properties.

Notably, a selective or more pronounced inhibition of soil AOB was recently determined for MHPP with AOA and AOB pure culture isolates (Kaur-Bhambra et al. 2022). Similarly, MHPP and MHPA had significantly higher efficacy (lower EC_50_ values) in the soil where the net nitrification potential was AOB/comammox driven (CS). This higher AOB/comammox-sensitivity to MHPP and MHPA, together with the significant positive correlation with the efficacy of the selective inhibitor octyne (Supplementary Fig. 8), suggest that these two BNIs are potentially selective AOB/comammox inhibitors. Nevertheless, further research on the inhibition patterns of pure ammonia oxidizer cultures by MHPA, and additional evidence of AOA-driven nitrification activity in the presence of MHPA and MHPP is required to confirm the selective inhibition of these BNIs (Taylor et al. 2013).

In addition to the degree of inhibition, chemical structure can also play a role in the mode of action or selectivity of NIs. It has been proposed that specific chemical structures favour inhibition of nitrification, and thus compounds with similar structures to known NIs are often expected to result in similar degrees of inhibition (White 1988, Subbarao et al. 2013). MHPP and MHPA are very similar phenylpropanoids, yet large differences in their EC_50_ values were observed (Fig. 5, Supplementary Table 4). In fact, similar differences in NI efficacies due to specific chemical structures have been observed previously. For example, a change in the configuration of one of the isomers of the NI brachialactone reduced its nitrification inhibition activity (Egenolf et al. 2020). Despite being an inhibitor of all known ammonia oxidizers, phenylacetylene inhibits AOA and AOB through a different mode of action (Wright et al. 2020). Previous studies have also shown that the degree of inhibition of *n*-alkynes, with different chain lengths, varies between AOA and AOB (Taylor et al. 2013, 2015, Wright et al. 2020). These observations indicate that besides the effect of soil pH, soil physicochemical properties, and the microbial community, the role of the chemical structure in NI efficacy is of relevance particularly amidst the complexity of natural environments such as soils.

## Conclusions

In this study we showed that several SNIs and BNIs are resilient to short-term soil pH changes, which indicates that besides soil pH, other soil physicochemical properties, the abundance and composition of ammonia oxidizers, and NI chemical structure play a role in the efficacy of NIs. Additionally, we showed the first evidence of two BNIs (MHPP and MHPA) as putative selective inhibitors and that MHPA and limonene inhibition was equal and, in some cases, superior to the inhibition caused by commonly used SNIs. Notably, the EC_80_ values of MHPA were similar to currently used DCD concentrations in agricultural soils (10% N applied), highlighting that some BNIs could be an effective alternative to SNIs. In summation, if BNIs are to be more widely adopted as a natural alternative to reduce N losses, it is key to understand the extent to which soil biotic and abiotic factors affect their efficacy to establish an optimal application rate in agricultural systems.

## Supplementary Material

fiae072_Supplemental_File
