# Supplementary material for: Inhibition profile of three biological nitrification inhibitors and their response to soil pH modification in two contrasting soils
Source: FEMS Microbiol Ecol. 2024 May 3;100(6):fiae072. doi: 10.1093/femsec/fiae072 (PMC11110862; doi:10.1093/femsec/fiae072)
Supplement: fiae072_Supplemental_File [file fiae072_supplemental_file.docx]

**Supplementary Table 1:** Reagents and qPCR conditions for AOA, AOB, ComB and ComA *amoA* genes

| Gene | Primer name | qPCR conditions | Primer final concentration | Reference |
| --- | --- | --- | --- | --- |
| AOA *amoA* | 104F  616R | 95°C for 15 min  40 cycles of:  95°C for 15 s  60°C for 45 s  78°C for 10 s  Final extension:  60°C for 10 min  Melting curve | 1 µM | Alves et al. 2013 |
| AOB *amoA* | 1F  2R | 94°C for 5 min  40 cycles of:  94°C for 1 min  60°C for 1 min 30 s  72°C for 1 min 30 s  Melting curve | 0.3 µM | Rotthauwe et al. 1997 |
| ComB *amoA* | comaB_244F  comaB_659R | 95°C for 3 min  40 cycles of:  95°C for 30 s  52°C for 45 s  72°C for 1 min  Melting curve | 0.5 µM | Pjevac et al. 2017 |
| ComA *amoA* | comaA_244F  comaA_659R | 95°C for 3 min  40 cycles of:  95°C for 30 s  52°C for 45 s  72°C for 1 min  Melting curve | 0.5 µM | Pjevac et al. 2017 |

For all genes, the mastermix consisted of 1X of the iQ SYBER Green supermix, between 0.1 and 0.2 mg/mL of BSA, forward and reverse primers at the respective final concentrations and 1µL of DNA template.

**Supplementary Table 2:** Significance (*P*) and explained variance (R^2^) of the environmental factors on the 16S rRNA, AOA, AOB and ComB *amoA* microbial communities. Environmental factors with a *P* < 0.01 that also contributed to a high explained variance are depicted in bold.

|  | Gene | | | | | | | |
| --- | --- | --- | --- | --- | --- | --- | --- | --- |
|  | 16S rRNA | | AOA *amoA* | | AOB *amoA* | | ComB *amoA* | |
|  | R^2^ | *P* | R^2^ | *P* | R^2^ | *P* | R^2^ | *P* |
| pH | 0.997 | **0.002** | 0.9986 | **0.002** | 0.9983 | **0.001** | 0.9968 | **0.003** |
| DOC | 0.989 | **0.003** | 0.9780 | **0.010** | 0.9864 | **0.002** | 0.9904 | **0.003** |
| TDN | 0.700 | 0.045 | 0.6134 | 0.052 | 0.7499 | 0.014 | 0.8032 | 0.027 |
| Cmic | 0.797 | 0.012 | 0.6153 | 0.039 | 0.7089 | 0.024 | 0.7933 | 0.033 |
| Ammonium | 0.780 | 0.040 | 0.7178 | 0.051 | 0.7144 | 0.023 | 0.8511 | 0.018 |
| Nitrate | 0.959 | 0.015 | 0.9450 | 0.014 | 0.9630 | 0.012 | 0.9531 | 0.023 |
| Total C | 0.965 | 0.020 | 0.9237 | 0.030 | 0.9533 | 0.019 | 0.9786 | **0.007** |
| CaCO3 | 0.988 | 0.015 | 0.9703 | 0.024 | 0.9802 | 0.015 | 0.9933 | **0.004** |
| Iron | 0.992 | **0.005** | 0.9940 | 0.011 | 0.9967 | **0.006** | 0.9900 | 0.016 |
| Manganese | 0.954 | **0.008** | 0.8841 | 0.019 | 0.9210 | 0.011 | 0.9624 | **0.007** |
| Copper | 0.701 | 0.055 | 0.5180 | 0.108 | 0.6495 | 0.048 | 0.7113 | 0.067 |
| CEC | 0.994 | **0.008** | 0.9930 | 0.012 | 0.9934 | 0.013 | 0.9943 | 0.015 |


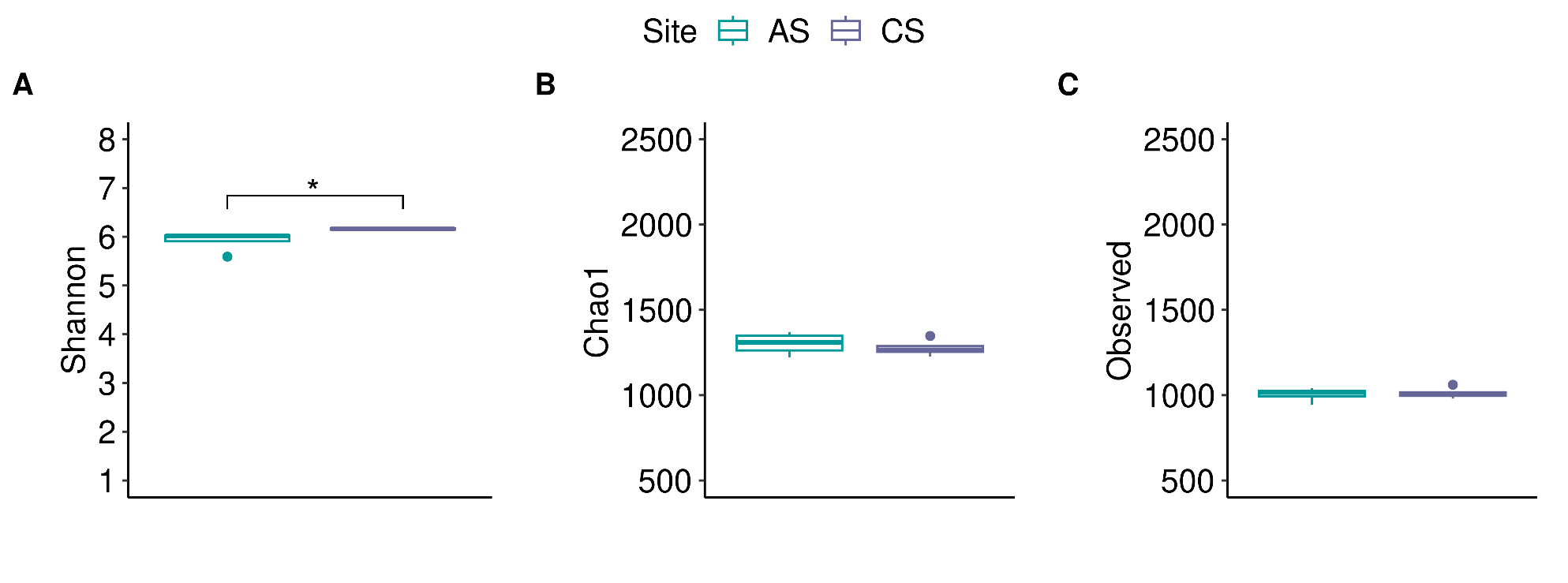


**Supplementary Figure 1:** α-diversity indexes of the 16S rRNA gene-based microbial community composition from the AS and CS. **A** Shannon diversity index, **B** Chao1 index, and **C** Observed ASVs richness, for the AS and the CS. Samples collected in spring 2022 (*n* = 4, * P < 0.05). The median value is depicted as the middle hinge in the boxplots. Upper and lower hinges represent the first and third quartile. The length of the whiskers is determined by the largest and the smallest value in the dataset that are within 1.5 times the inter-quartile range. Points outside the length of the whiskers are depicted individually.


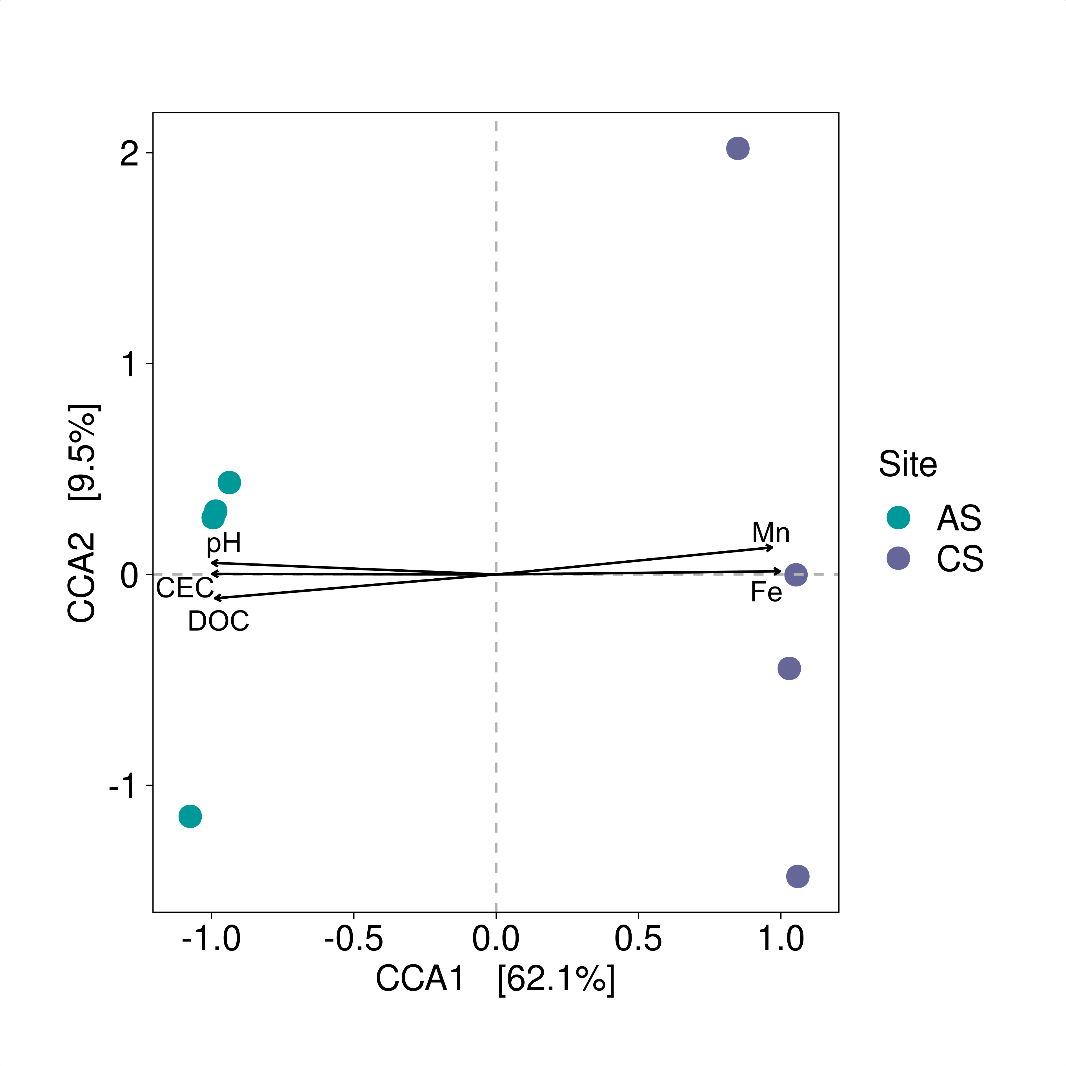


**Supplementary Figure 2:** Soil microbial community β-diversity in the alkaline (AS) and the circumneutral soil (CS). Canonical correspondence analysis on a Bray-Curtis dissimilarity matrix of the 16S rRNA microbial communities in the AS and CS from samples collected in spring 2022 (*n* = 4).


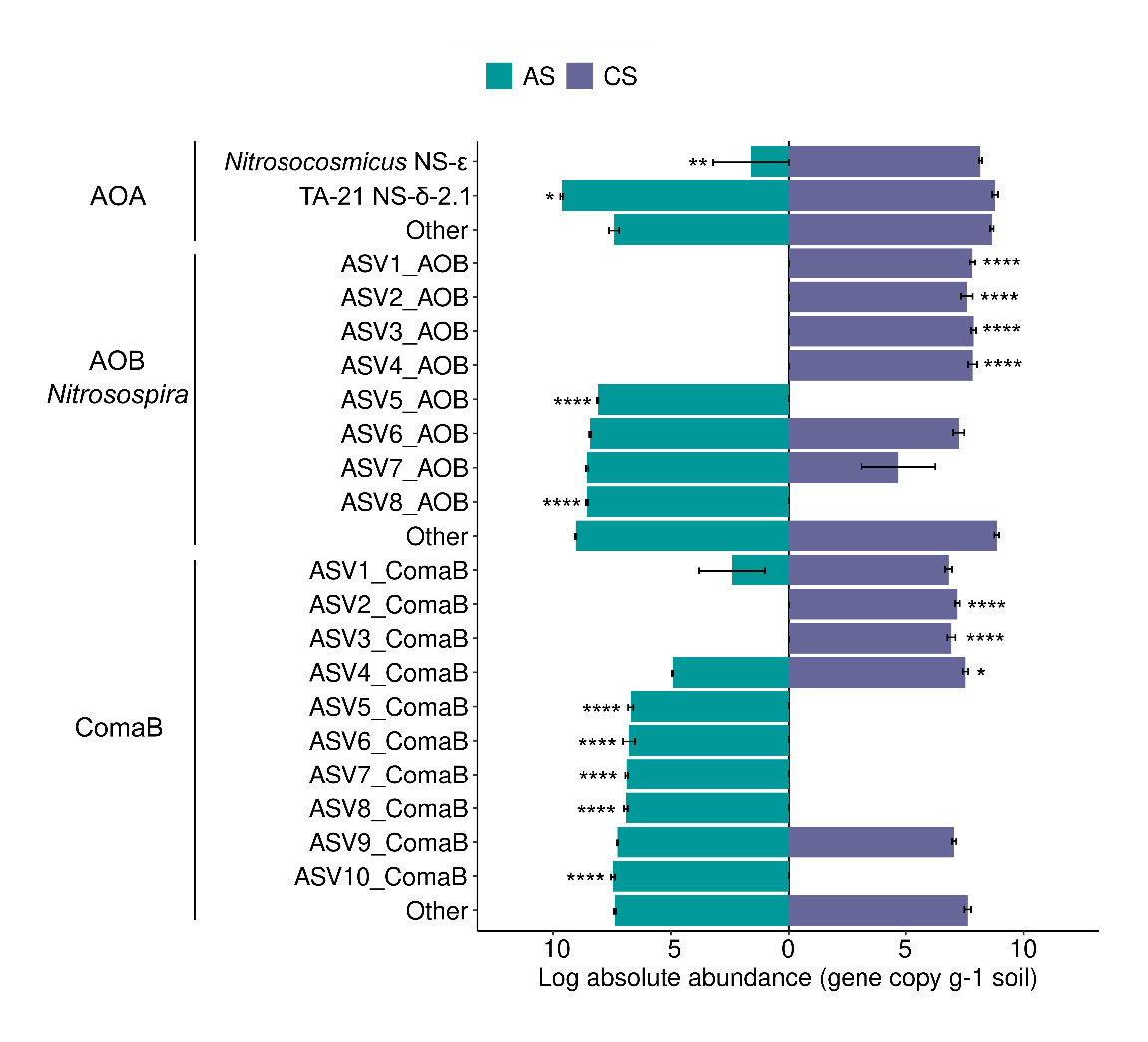


**Supplementary Figure 3:** Absolute abundance of the top *amoA* taxa with 5% mean relative abundance. Samples collected in spring 2022 (*n* = 4, * P < 0.05, **P < 0.01, ***P < 0.001, ****P < 0.0001).


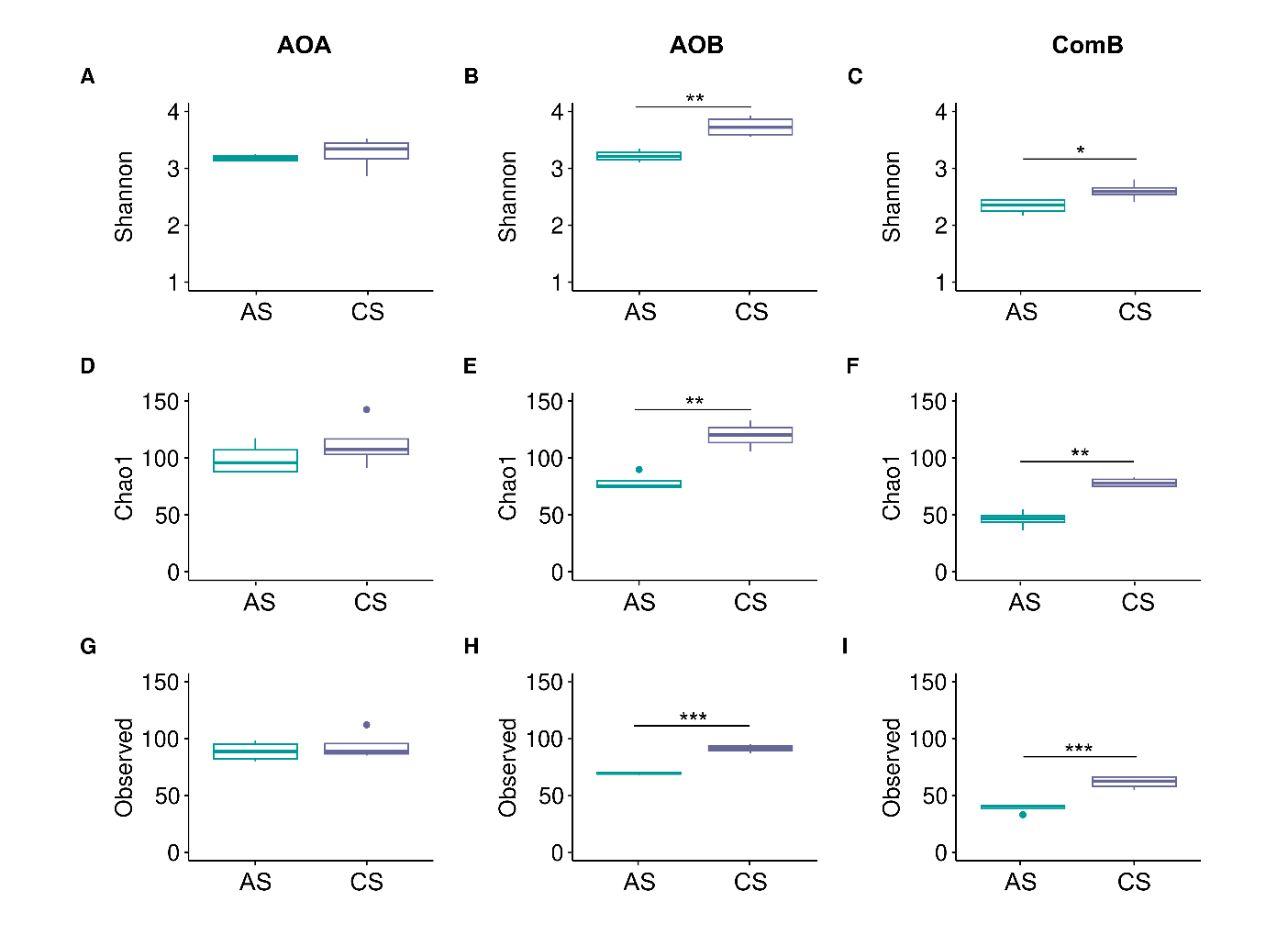


**Supplementary Figure 4:** α-diversity of the ammonia oxidizer microbial community composition from the AS and CS. **A**, **B** and **C** Shannon diversity index **D**, **E**, **F** Chao1 index **G**, **H**, **I** Observed ASVs richness for the AOA, AOB and ComB communities in the AS and CS. Samples collected in spring 2022 (*n* = 4). The median is depicted as the middle hinge in the boxplots. Upper and lower hinges represent the first and third quartile. The length of the whiskers is determined by the largest and the smallest value in the dataset that are within 1.5 times the inter-quartile range. Points outside the length of the whiskers are depicted individually. (* P < 0.05, ** P < 0.01, *** P < 0.001).


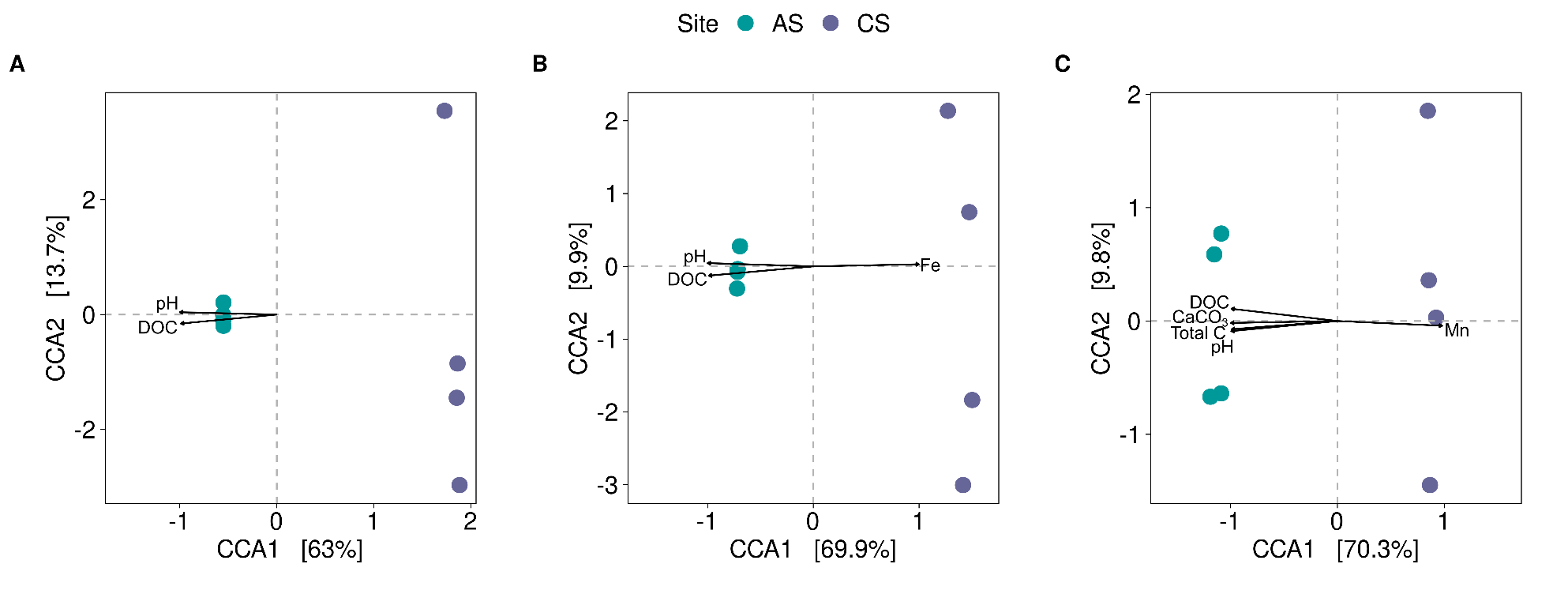


**Supplementary Figure 5:** Canonical correspondence analysis on a Bray-Curtis dissimilarity matrix of the ammonia oxidizer communities in the AS and CS from samples collected spring 2022 (*n* = 4) **A** AOA. **B** AOB. **C** ComB.


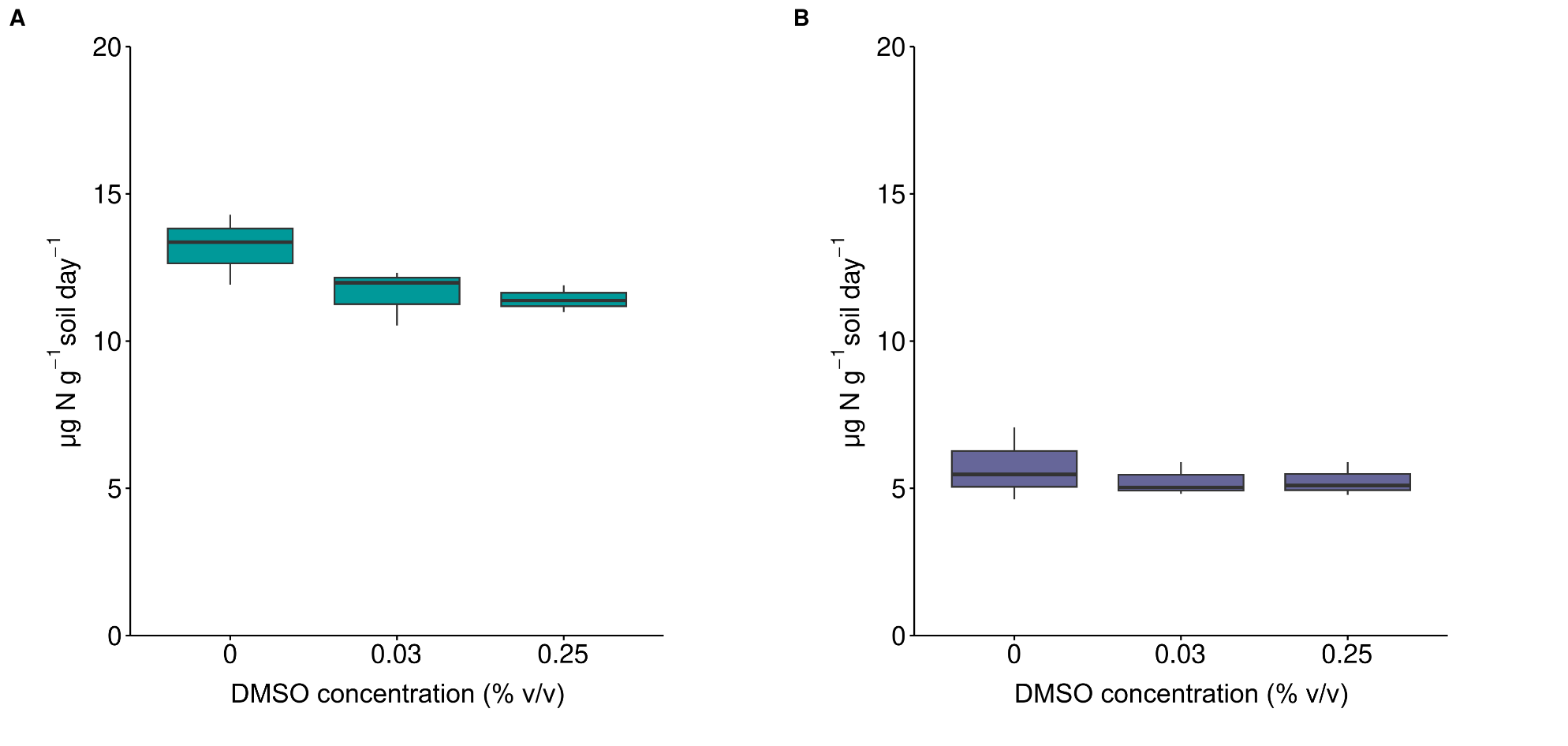


**Supplementary Figure 6:** Effect of DMSO on the net nitrification potential. Net nitrification potential **A** in the AS and **B** in the CS supplemented with different concentrations of DMSO. One-way ANOVA resulted in not significant differences among the different DMSO concentrations (P = 0.106 for the AS and P = 0.747 for the CS). The median is depicted as the middle hinge in the boxplots. Upper and lower hinges represent the first and third quartile. The length of the whispers is determined by the largest and the smallest value in the dataset that are within 1.5 times the inter-quartile range (*n* = 3).


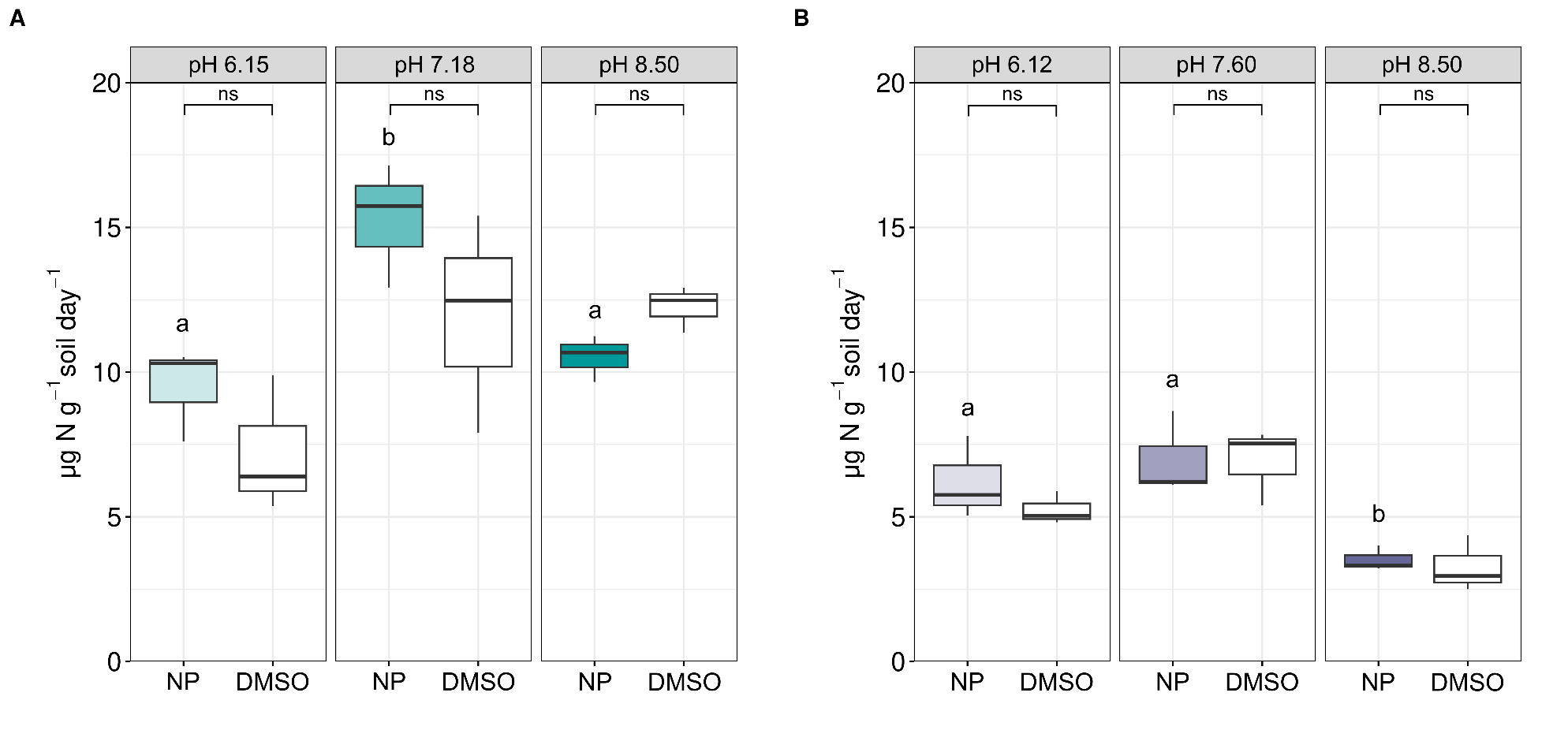


**Supplementary Figure 7:** Effect of soil pH modification on the net nitrification potential activity in the AS (pH 8.5) and CS (pH 6.12). **A** Net nitrification potential (NP) and the respective DMSO controls at pH: 6.15, 7.18, and 8.50 in the AS and **B** at pH: 6.12, 7.60, and 8.50 in the CS. Lower case letters depict significant differences in the NP at the different pHs in each soil. Not significant (ns) differences were observed between the NP at each pH and the respective DMSO control regardless of soil. The median is depicted as the middle hinge in the boxplots. Upper and lower hinges represent the first and third quartile. The length of the whiskers is determined by the largest and the smallest value in the dataset that are within 1.5 times the inter-quartile range. Samples collected in spring 2022 (*n* = 3).


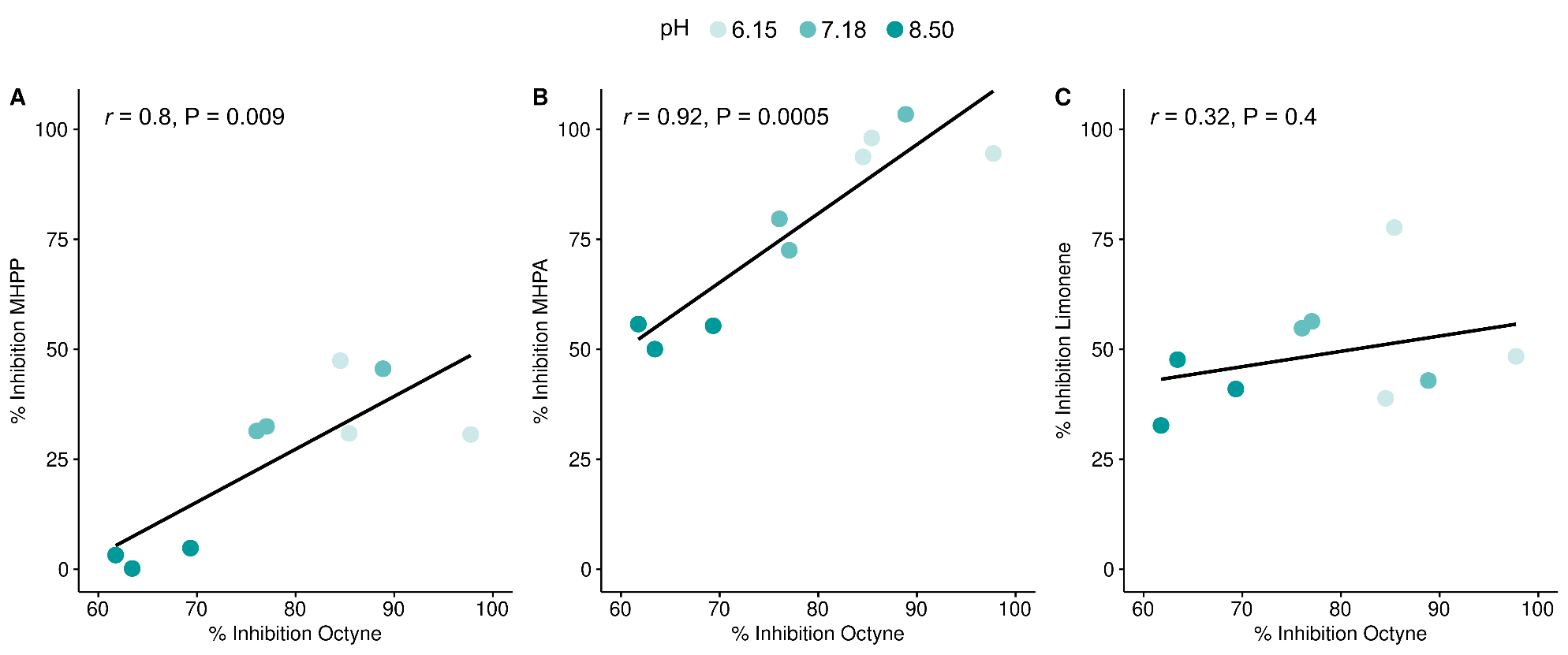


**Supplementary Figure 8:** Correlation between the efficacy of the known selective inhibitor, octyne and three BNIs at the different pH modifications carried out in the AS. Correlations between octyne and two putative selective inhibitors: **A** MHPP and **B** MHPA. **C** Correlation between the efficacies of octyne and limonene. Samples collected in spring 2022 (*n* = 3).

**Supplementary Table 3:** Pair-waise comparisons of the EC_50_ and EC_80_ values of MHPP, MHPA and limonene in the AS and CS. Significant differences are depicted in bold based on a two-tailed t-test with an α of 0.05.

| Effective concentration | Comparison | Means difference | Pooled standard error | Calculated t-value | Critical t-value | Degrees of freedom |
| --- | --- | --- | --- | --- | --- | --- |
| EC_50_ | MHPP (AS) vs MHPP (CS) | 100.9 | 36.7 | **2.7** | **2.04** | 31 |
|  | MHPA (AS) vs MHPA (CS) | 68.7 | 14.7 | **4.6** | **2.04** | 31 |
|  | Limonene (AS) vs Limonene (CS) | 5.8 | 41.0 | 0.1 | 2.08 | 21 |
|  | MHPP (AS) vs MHPA (AS) | 356.8 | 33.2 | **10.7** | **2.04** | 34 |
|  | MHPP (AS) vs Limonene (AS) | 357.6 | 41.8 | **8.5** | **2.04** | 28 |
|  | MHPA (AS) vs Limonene (AS) | 0.7 | 29.1 | 0.02 | 2.04 | 28 |
|  | MHPP (CS) vs MHPA (CS) | 324.6 | 21.4 | **15.1** | **2.04** | 28 |
|  | MHPP (CS) vs Limonene (CS) | 262.5 | 35.7 | **7.3** | **2.06** | 24 |
|  | MHPA (CS) vs Limonene (CS) | -62.1 | 32.3 | -1.9 | 2.06 | 24 |
| EC_80_ | MHPP (AS) vs MHPP (CS) | 158.5 | 94.7 | 1.6 | 2.04 | 31 |
|  | MHPA (AS) vs MHPA (CS) | 40.1 | 50.4 | 0.7 | 2.04 | 31 |
|  | Limonene (AS) vs Limonene (CS) | 510.9 | 402.6 | 1.2 | 2.08 | 21 |
|  | MHPP (AS) vs MHPA (AS) | 644 | 84.8 | **7.5** | **2.04** | 34 |
|  | MHPP (AS) vs Limonene (AS) | -35.1 | 371.4 | -0.09 | 2.04 | 28 |
|  | MHPA (AS) vs Limonene (AS) | -679.2 | 363.0 | -1.8 | 2.04 | 28 |
|  | MHPP (CS) vs MHPA (CS) | 525.6 | 65.8 | **7.9** | **2.04** | 28 |
|  | MHPP (CS) vs Limonene (CS) | 317.1 | 182.0 | 1.7 | 2.06 | 24 |
|  | MHPA (CS) vs Limonene (CS) | -208.4 | 181.3 | -1.1 | 2.06 | 24 |


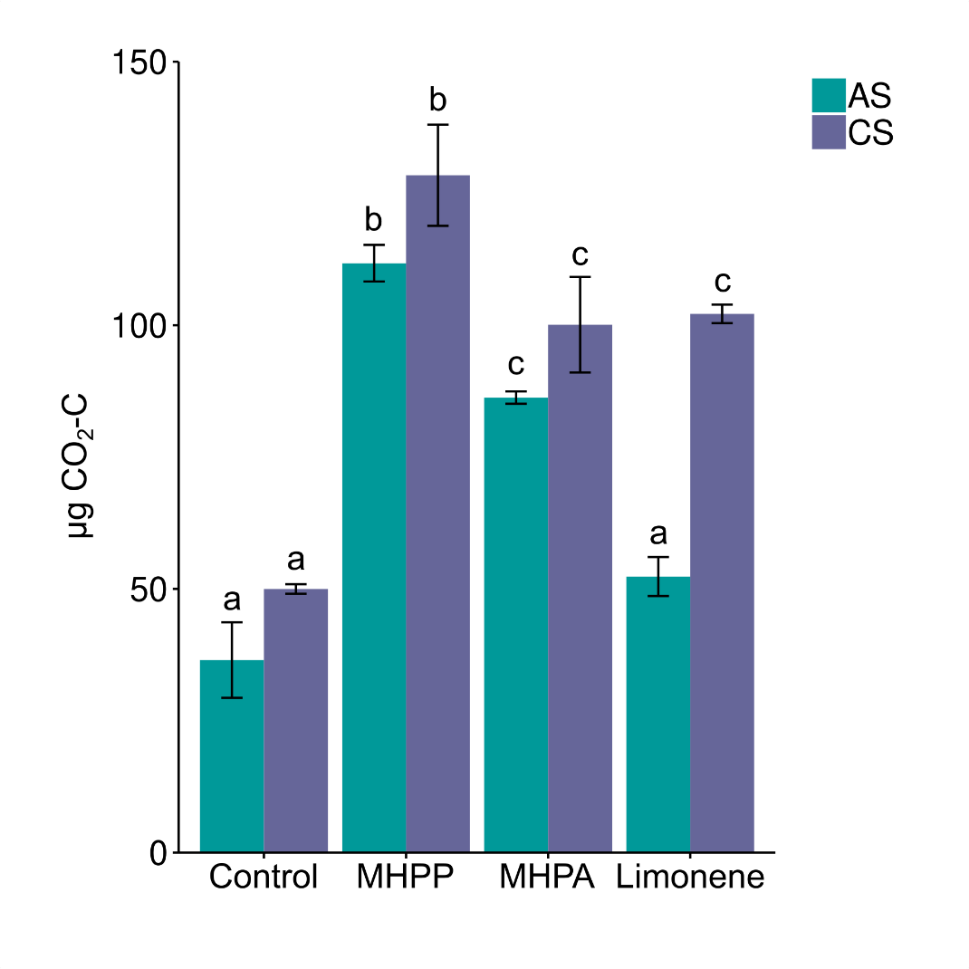


**Supplementary Figure 9:** CO_2_-C production in the AS and CS. The µg CO_2_-C produced in the control without inhibitor and in the amendments with the three BNIs tested in the AS and CS are depicted. Significant differences are depicted in lower case letters. Error bars represent the standard error in each case. Samples collected in spring 2022 (*n* = 4, P < 0.05).

**Supplementary Table 4:** List of synthetic and biological nitrification inhibitors used in this study.

|  | Compound name | Compound type | Origin | Mode of action | Enzyme target | Molecular structure | Ref. |
| --- | --- | --- | --- | --- | --- | --- | --- |
| BNIs | Methyl 3-(4-hydroxyphenyl) propionate (MHPP) | Phenylpropanoid | *Sorghum bicolor* | Unknown | AMO |  | (Zakir et al. 2008; Beeckman et al. 2023) |
|  | Methyl 3-(4-hydroxyphenyl) acrylate (MHPA) | Phenylpropanoid | *Brachiaria*  *humidicola* | Unknown | Unknown |  | (Gopalakrishnan et al. 2007; Ghatak et al. 2023) |
|  | Limonene | Monoterpene | *Pinus*  *ponderosa* | Unknown | AMO |  | (White 1991; Nardi et al. 2020; Ghatak et al. 2023) |
| SNIs | Dicyandiamide (DCD) | Aminocarbox-amidine | Synthetic | Cu-chelator | AMO |  | (Zerulla et al. 2001; Nardi et al. 2020; Beeckman et al. 2023) |
|  | 3,4-Dimethylpyrazole phosphate  (DMPP) | Pyrazol | Synthetic | Cu-chelator | AMO |  | (Zerulla et al. 2001; Beeckman et al. 2023) |
|  | Nitrapyrin | Chloropyridine | Synthetic | Binding to Cu site | AMO |  | (Powell and Prosser 1985; Vannelli and Hooper 1992) |
| SNIs for laboratory studies | Phenylacetelyne | Alkyne | Synthetic | Non-competitive inhibitor | AMO |  | (McCarty and Bremner 1986) |
|  | 1-Octyne | Alkyne | Synthetic | Competitive inhibitor | AMO |  | (Taylor et al. 2013, 2015) |
|  | Allylthiourea (ATU) | Thiourea | Synthetic | Cu- chelator | AMO |  | (Bédard and Knowles 1989) |
